# Effects of circulating inflammatory proteins on spinal degenerative diseases: Evidence from genetic correlations and Mendelian randomization study

**DOI:** 10.1002/jsp2.1346

**Published:** 2024-06-17

**Authors:** Qingcong Zheng, Rongjie Lin, Du Wang, Chunfu Zheng, Weihong Xu

**Affiliations:** ^1^ Department of Spinal Surgery The First Affiliated Hospital of Fujian Medical University Fuzhou China; ^2^ Department of Orthopedic Surgery Fujian Medical University Union Hospital Fuzhou China; ^3^ Arthritis Clinical and Research Center, Peking University People's Hospital Beijing China; ^4^ Department of Microbiology, Immunology and Infectious Diseases University of Calgary Calgary Alberta Canada

**Keywords:** cervical spondylosis, circulating inflammatory proteins, Mendelian randomization, prolapsed disc/slipped disc, spinal canal stenosis, spinal degenerative diseases, spondylolisthesis/spondylolysis

## Abstract

**Background:**

Numerous investigations have suggested links between circulating inflammatory proteins (CIPs) and spinal degenerative diseases (SDDs), but causality has not been proven. This study used Mendelian randomization (MR) to investigate the causal associations between 91 CIPs and cervical spondylosis (CS), prolapsed disc/slipped disc (PD/SD), spinal canal stenosis (SCS), and spondylolisthesis/spondylolysis.

**Methods:**

Genetic variants data for CIPs and SDDs were obtained from the genome‐wide association studies (GWAS) database. We used inverse variance weighted (IVW) as the primary method, analyzing the validity and robustness of the results through pleiotropy and heterogeneity tests and performing reverse MR analysis to test for reverse causality.

**Results:**

The IVW results with Bonferroni correction indicated that beta‐nerve growth factor (β‐NGF), C‐X‐C motif chemokine 6 (CXCL6), and interleukin‐6 (IL‐6) can increase the risk of CS. Fibroblast growth factor 19 (FGF19), sulfotransferase 1A1 (SULT1A1), and tumor necrosis factor‐beta (TNF‐β) can increase PD/SD risk, whereas urokinase‐type plasminogen activator (u‐PA) can decrease the risk of PD/SD. FGF19 and TNF can increase SCS risk. STAM binding protein (STAMBP) and T‐cell surface glycoprotein CD6 isoform (CD6 isoform) can increase the risk of spondylolisthesis/spondylolysis, whereas monocyte chemoattractant protein 2 (MCP2) and latency‐associated peptide transforming growth factor beta 1 (LAP‐TGF‐β1) can decrease spondylolisthesis/spondylolysis risk.

**Conclusions:**

MR analysis indicated the causal associations between multiple genetically predicted CIPs and the risk of four SDDs (CS, PD/SD, SCS, and spondylolisthesis/spondylolysis). This study provides reliable genetic evidence for in‐depth exploration of the involvement of CIPs in the pathogenic mechanism of SDDs and provides novel potential targets for SDDs.

## Graphical abstract

MR analyses indicated the causal associations between multiple genetically predicted CIPs and the risk of four SDDs (CS, PD/SD, SCS, and spondylolisthesis/spondylolysis). This study provides reliable genetic evidence for in‐depth exploration of the involvement of CIPs in the pathogenic mechanism of SDDs and provides novel potential targets for SDDs.

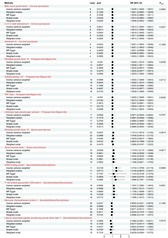

## 1 INTRODUCTION

Spinal degenerative diseases (SDDs) are a group of age‐related diseases characterized by degeneration of the intervertebral disc, vertebral bone, and its accessory structures.^1^ SDDs are the primary cause of low back pain (LBP) and neck pain (NP), and the Global Burden of Diseases study indicated that LBP significantly contributes to years lived with disability (YLDs) globally.^2^ The prevalence of SDDs is increasing under the trend of an aging society, and SDDs are not only responsible for a large number of labor force losses and disabilities globally but also pose a huge economic burden.^3^ The most prevalent kinds of SDDs are cervical spondylosis (CS), prolapsed disc/slipped disc (PD/SD), spinal canal stenosis (SCS), and spondylolisthesis/spondylolysis. The major pathological underpinning for these conditions is intervertebral disc (IVD) degeneration (IVDD) and imbalanced bone homeostasis.^4^ Despite the amount of time and money spent on research into SDDs, a cure is still needed due to the extraordinarily complex pathogenic mechanisms.

Currently, it is thought that a combination of aging, supraphysiological loads, and genetics causes SDDs,^5^ and the primary pathogenic processes of SDDs include oxidative stress, mitochondrial dysfunction, extracellular matrix (ECM) breakdown, cell death, and inflammation.^6^ It has been shown that nucleus pulposus (NP) cell senescence can release senescence‐associated secretory phenotype (SASP) represented by inflammatory factors and lead to further senescence of itself and surrounding cells.^7^ Hyperphysiological mechanical loading of the IVD, either static or dynamic, leads to upregulation of interleukin‐1 beta (IL‐1β) and tumor necrosis factor‐alpha (TNF‐α) levels and apoptosis.^8^ Oxidative stress and mitochondrial dysfunction can induce disc cells to promote inflammation and intensify oxidative stress through mitogen‐activated protein kinase (MAPK) and nuclear factor‐kappa B (NF‐κB) signaling pathways, creating a vicious cycle.^9^ It has been proposed that the basic pathophysiology of IVDD is inflammation brought on by an unbalanced immunological microenvironment,^10^ in which circulating inflammatory proteins (CIPs) are essential. Certain CIPs can cause IVD cells to senescence and apoptosis, activate proteolytic enzymes to increase extracellular matrix (ECM) degradation, and aid in the infiltration of immune cells to amplify the inflammatory response, all of which can lead to the formation of SDDs.^11^ However, by suppressing senescence and SASP production, downregulating pro‐inflammatory cytokines and chemokines to avoid inflammation, and encouraging repair and regeneration through the deposition of IVD‐important proteins, other CIPs can postpone the development of IVDD.^12^, ^13^ Reports on the association between CIPs and the onset of SDDs are controversial, and bias due to the combination of confounding factors, environmental factors, and reverse causation is one of the main reasons why these studies are contradictory. A great deal of work has been done on the interconnections between CIPs and the multiple pathogenic mechanisms of SDDs. However, exploring their causal associations in terms of genetic factors has yet to be clarified.

Mendelian randomization (MR) is an epidemiological research strategy that uses effective instrumental variables (IVs) to analyze summary‐level data from the genome‐wide association studies (GWAS) data. Due to the stochastic nature of genetic variation and the fact that alleles are not affected by the environment of the disease, MR analysis can greatly reduce the bias caused by confounding factors and environmental factors and can consistently and reliably infer the causal association between exposure and outcome.^14^ Randomized controlled trials (RCTs) are a scientific research methodology that evaluates the effect of a causative factor or treatment regimen on a disease after a certain period by randomly assigning interventions to participants. RCTs are the gold standard for exploring the causal impact of exposure factors/interventions on disease.^15^ However, the implementation of RCTs requires significant workforce, time and funding. The issues of sample size, intervention duration and ethics also limit the effective implementation of RCTs.^16^ Although the strength of research evidence for MR analysis is weaker than for RCTs, it is stronger than for observational studies. MR analysis can provide RCTs with evidence of causal inference between risk factors and disease, further clarify research directions and potential therapeutic targets, and reduce the workforce and economic burden.^17^ Therefore, utilizing a bidirectional MR method for 91 CIPs and 4 SDDs (CS, PD/SD, SCS, and spondylolisthesis/spondylolysis), our study investigated if there is a genetically predicted causal association between exposures and outcomes.

## 2 MATERIALS AND METHODS

### 2.1 Study design

With the guidance of “strengthening the reporting of observational studies in epidemiology using Mendelian randomization (STROBE‐MR)”, we completed this study.^18^ The datasets used were obtained from publicly available GWAS databases and did not require ethical approval. Single nucleotide polymorphisms (SNPs) used as reliable IVs in MR analyses must fulfill the following three key assumptions. (1) The relevance assumption, the IVs must be strongly and directly related to exposure; (2) the independence assumption, the IVs have no association with the potential confounders; and (3) the exclusion restriction assumption: the IVs only affect outcome via the exposure pathway (Figure S1).

### 2.2 GWAS data sources

The summary‐level statistics for all cases and controls in this study, whether CIPs or SDDs, were derived from European ancestry, with the aim of reducing bias due to race‐related confounders. *Exposure data sources*. We searched 91 CIPs datasets (accession numbers from GCST90274758 to GCST90274848) from GWAS (https://www.ebi.ac.uk/gwas/home).^19^ *Outcome data sources*. We searched the CS dataset (accession number is “ukb‐b‐2349”), the PD/SD dataset (accession number is “ukb‐b‐15 904”), the SCS dataset (accession number is “ebi‐a‐GCST90018922”), and the spondylolisthesis/spondylolysis dataset (accession number is “finn‐b‐M13_SPONDYLOLISTHESIS”) from Integrative Epidemiology Unit (IEU, https://gwas.mrcieu.ac.uk/). After comparing the sources of participants in the 91 datasets from the CIPs with the 4 datasets from the SDDs, we considered that the samples of the GWAS data for exposures and outcomes were independent of each other as a way of reducing bias due to overlapping data sources. Details of these data sources are given in Table S1.

### 2.3 Selection of instrumental variables

Identifying valid IVs is a critical first step in MR analysis. We screened for SNPs suggesting causal associations between exposures and outcomes by setting a “significance threshold of 5E‐06” in the 91 CIPs dataset. The extracted SNPs were analyzed by the “clump_data” function for linkage disequilibrium analysis at “*r* ^2^ < 0.001, 10 000 kb”. The purpose of this step was to exclude mutual linkage SNPs and to discard non‐biallelic SNPs as a way to ensure independence among IVs for each exposure. We used *F*‐statistic to assess the strength of the association between the screened IVs and exposure, with the aim of avoiding bias caused by weak IVs. The *F*‐statistic is calculated as *F* = (*β* ^2^/standard error^2^) when *F* > 10 for SNPs indicates that it is strong IVs.^20^ When the effect alleles for the SNPs' effects of exposure and outcome are different, the summary set may be incorrect. Therefore, we used the “harmonise_data” function to test the causal direction of the screened SNPs in exposure, and the outcome eliminated palindromic alleles, and finally selected SNPs with a result of “TRUE” as valid IVs.

### 2.4 Two‐sample Mendelian randomization (TSMR) analysis

Our study was based on the analysis of GWAS data with the “TwoSampleMR” package of the R version 4.2.3 software. Forward MR was analyzed with 91 CIPs as exposures and CS, PD/SD, SCS, and spondylolisthesis/spondylolysis as outcomes. Reverse MR, in which exposure and outcome were interchanged, was performed with the aim of excluding bias due to reverse causality. First, to assess the causal associations between exposures and outcomes, we chose MR Egger, weighted median, random effects IVW, simple mode, and weighted mode methods, of which IVW is the most valid and reliable. We applied the Bonferroni correction to the results using the “p.adjust” function in the R software and recorded objects with *P*_adjust_ < 0.2. The results of IVs, all‐MR Egger, and all‐IVW were visualized by forest plot. Second, heterogeneity analysis. Cochran's Q‐statistic was used to detect the heterogeneity of SNPs in IVW and MR‐Egger analyses as a means of assessing the robustness of IVs, and when *p* < 0.05 indicates that the results are heterogeneous.^21^ The results of the heterogeneity test were visualized by funnel plots. Third, pleiotropy analyses. Pleiotropy is the fact that some IVs can influence outcomes through confounding factors other than exposure, which would seriously affect the reliability of the causal association between exposure and outcome. MR‐PRESSO can detect the outlier and give the causal change in exposure and outcome after excluding the outlier.^22^ In addition, we performed effect estimation and bias detection using the MR‐Egger intercept, and when the *p*‐value calculated by the ‘MR_pleiotropy_test’ function greater than 0.05, it showed no evidence of directional pleiotropy.^23^ Leave‐one‐out analysis refers to the method of assessing the effect of the remaining SNPs on the outcome by performing IVW analyses after removing individual SNPs in sequential order, with the aim of discovering whether there are any single SNPs driving causality.

## 3 RESULTS

### 3.1 Forward MR

**FIGURE 1:**
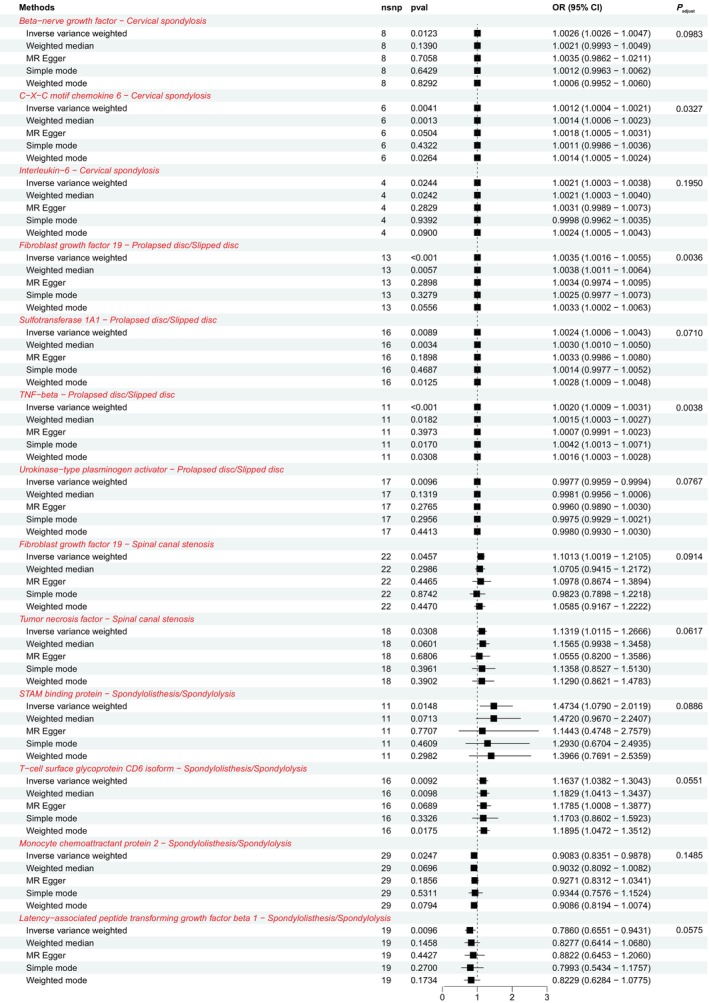
Summary forest plot showed the causal associations between CIPs and SDDs (cervical spondylosis, prolapsed disc/slipped disc, spinal canal stenosis, and spondylolisthesis/spondylolysis) in the forward MR analysis. SNP, single‐nucleotide polymorphism; OR, odds ratio; 95% CI, 95% confidence interval.

We identified 3, 4, 2, and 4 CIPs causally associated with CS, PD/SD, SCS, and spondylolisthesis/spondylolysis, respectively. The MR and the sensitivity analysis results are shown in Tables S2–S5, the detailed information on the IVs is presented in Tables S6–S9, and the summarized results are shown in the forest plot (Figure 1). The SNPs used as IVs all had strong effects (*F*‐statistic >10). IVW analysis revealed that beta‐nerve growth factor (β‐NGF) (OR = 1.0026; 95% CI: 1.0026–1.0047; *p* = 0.01223; *P*_adjust_ = 0.0983), C‐X‐C motif chemokine 6 (CXCL6) (OR = 1.0012; 95% CI: 1.0004–1.0021; *p* = 0.0041; *P*_adjust_ = 0.0327), and interleukin‐6 (IL‐6) (OR = 1.0021; 95% CI: 1.0003–1.0038; *p* = 0.0244; *P*_adjust_ = 0.1950) can increase the risk of CS (Figure S2). Fibroblast growth factor 19 (FGF19) (OR = 1.0035; 95% CI: 1.0016–1.0055; *p* = 0.0004; *P*_adjust_ = 0.0036), sulfotransferase 1A1 (SULT1A1) (OR = 1.0024; 95% CI: 1.0006–1.0043; *p* = 0.0089; *P*_adjust_ = 0.0710), and tumor necrosis factor‐beta (TNF‐β) (OR = 1.0020; 95% CI: 1.0009–1.0031; *p* = 0.0005; *P*_adjust_ = 0.0038) can increase PD/SD risk, whereas urokinase‐type plasminogen activator (u‐PA) (OR = 0.9977; 95% CI: 0.9959–0.9994; *p* = 0.0096; *P*_adjust_ = 0.0767) can reduce PD/SD risk (Figure S3). FGF19 (OR = 1.1013; 95% CI: 1.0019–1.2105; *p* = 0.0457; *P*_adjust_ = 0.0914) and TNF (OR = 1.1319; 95% CI: 1.0115–1.2666; *p* = 0.0308; *P*_adjust_ = 0.0617) can increase the risk of SCS (Figure S4). STAM binding protein (STAMBP) (OR = 1.4734; 95% CI: 1.0790–2.0119; *p* = 0.0148; *P*_adjust_ = 0.0886), and T‐cell surface glycoprotein CD6 isoform (CD6 isoform) (OR = 1.1637; 95% CI: 1.0382–1.3043; *p* = 0.0092; *P*_adjust_ = 0.0551) can increase spondylolisthesis/spondylolysis risk, whereas monocyte chemoattractant protein 2 (MCP2) (OR = 0.9083; 95% CI: 0.8351–0.9878; *p* = 0.0247; *P*_adjust_ = 0.1485) and latency‐associated peptide transforming growth factor beta 1 (LAP‐TGF‐β1) (OR = 0.7860; 95% CI: 0.6551–0.9431; *p* = 0.0096; *P*_adjust_ = 0.0575) can reduce spondylolisthesis/spondylolysis risk (Figure S5). We visualized the results of IVs, all‐MR Egger, and all‐IVW by forest plot (Figures S6–S9). In sensitivity analyses, the p‐values of the pleiotropy test (MR‐Egger intercept test and MR‐PRESSO global test) and heterogeneity test (Cochran's *Q*‐statistic) for these 13 causal associations were all greater than 0.05. SNPs were symmetrically distributed in the funnel plot of IVW in the above results (Figures S10–S13). As can be seen from the leave‐one‐out plot, no single SNP had an obvious influence on the association (Figures S14–S17). Thus, the sensitivity analysis showed no significant pleiotropy and heterogeneity in the results, and the MR analysis had good validity and robustness.

### 3.2 Reverse MR

We list the results of reverse MR and sensitivity analyses between SDDs and CIPs in Tables S10–S13 and the detailed information on IVs in Tables S14–S17. The SNPs used as IVs all had strong effects (*F*‐statistic >10). IVW analyses showed that there was no causal effect of CS on the levels of β‐NGF (*p* = 0.0681), CXCL6 (*p* = 0.1495), and IL‐6 (*p* = 0.6636) (Figure S18). PD/SD had no causal effect on the levels of FGF19 (*p* = 0.0558), SULT1A1 (*p* = 0.1908), and TNF‐β (*p* = 0.2364) (Figure S19). SCS had no causal effect on the levels of FGF19 (*p* = 0.1952) and TNF (*p* = 0.0931) (Figure S20). Spondylolisthesis/spondylolysis also had no causal effect on the levels of STAMBP (*p* = 0.5197), CD6 isoform (*p* = 0.7240), MCP2 (*p* = 0.5843), and LAP‐TGF‐β1 (*p* = 0.0879) (Figure S21). In the MR analysis of PD/SD on u‐PA, there was no evidence to support a causal association between them due to the presence of pleiotropy (MR‐Egger intercept *p*‐value <0.05) (Table S11). In the MR analysis of PD/SD on TNF‐β (Table S11), spondylolisthesis/spondylolysis on STAMBP and CD6 isoform (Table S13), although the Cochran's Q *p*‐value for IVW <0.05 indicated the presence of heterogeneity, the heterogeneity was considered acceptable in this study using random effects IVW.^24^ In conclusion, the reverse MR results indicate that there is no obvious bias caused by reverse causality in forward MR. The reverse MR analysis of forest plots (Figures S22–S25), funnel plots (Figures S26–S29), and leave‐one‐out plots (Figures S30–S33) is shown in Supplementary Material.

## 4 DISCUSSION

We were the first to use MR analysis to investigate the causal associations between 91 CIPs and 4 SDDs. We found that genetically predicted β‐NGF, CXCL6, and IL‐6 increased the risk of CS; FGF19, SULT1A1, and TNF‐β increased the risk of PD/SD, whereas u‐PA decreased the risk of PD/SD; FGF19 and TNF increased the risk of SCS; and MCP2 and LAP‐TGF‐β1 reduced the risk of spondylolisthesis/spondylolysis, whereas STAMBP and CD6 isoform raised the risk. The results mentioned above are not inversely causal.

### 4.1 Associations of CIPs with CS

The nerve growth factor was the first neurotrophin to be characterized and can regulate neuronal survival.^25^ There have been reports indicating that β‐NGF can reduce neuron damage.^26^ Nevertheless, there is evidence to suggest that β‐NGF is more effective in inducing pain and neuroinflammation. β‐NGF not only promotes leukocyte infiltration, leading to the release of histamine from mast cells but also promotes the release of neurotransmitters from damaged neurons to trigger inflammatory pain.^27^ In bone homeostasis, β‐NGF can promote the transformation of cartilage to bone to increase bone mineral density (BMD) and play a mineralizing role.^28^ In addition, high expression of β‐NGF was found in an experiment in which annulus fibrosus cells were co‐cultured with neuron‐like cells, and β‐NGF can cause intraneural growth as well as inflammatory responses that contribute to the development of IVDD.^29^ Our study discovered that β‐NGF facilitates the formation of CS, offering a precise genetic foundation for therapeutic intervention. Granulocyte chemoattractant protein‐2 (GCP‐2) is another name for CXCL6, a pro‐inflammatory cytokine that can increase inflammation by drawing neutrophils.^30^ It has been shown that circulating CXCL6 levels were significantly increased in patients with IVDD, and CXCL6 can be used as a biomarker for IVDD.^31^ CXCL6 expression was also detected in a study simulating the IVDD environment.^32^ This study found that CXCL6 is a risk factor for encouraging CS. IVDD is an inflammatory condition, and one of its key characteristics is an elevation of the pro‐inflammatory cytokine IL‐6.^33^ IL‐6 not only promotes TNF‐α expression to enhance NP cell catabolism, promotes neuronal apoptosis, and induces hyperalgesia but also accelerates the process of IVDD through an inflammatory cascade, and targeting IL‐6 can be an effective treatment.^11^, ^34^ Other studies have reported that IL‐6 is a stress‐sensitive cytokine and that increased mechanical loading leads to IL‐6 secretion by osteoblasts, which promotes bone remodeling.^35^ According to this study, IL‐6 may raise the chance of developing CS. In summary, β‐NGF, CXCL6, and IL‐6 may be useful targets for CS treatment.

### 4.2 Associations of CIPs with PD/SD and SCS

The most varied family of growth factors, fibroblast growth factors (FGFs), are crucial for controlling a number of processes, including cell survival and death, proliferation and arrest, metabolism, and differentiation.^36^ IVDD is closely associated with the destruction of cartilage tissue, and the role of FGFs in IVD and cartilage is complex and variable.^37^ For example, FGF2 can increase the number of NP cells to delay the development of IVDD,^38^ and FGF6 and FGF7 can achieve the regenerative effect of IVD by inducing chondrocyte proliferation and promoting ECM remodeling.^39^ However, FGF1 can promote cartilage degradation in ways such as upregulating matrix metalloproteinase levels.^36^, ^40^ The FGFs family is crucial for maintaining IVD and cartilage homeostasis.^41^ Among them, FGF19 is a therapeutic target that has attracted much attention in the skeletomuscular system in recent years, and this study found that FGF19 not only promotes PD/SD onset but also increases the risk of SCS. FGF19 can reduce DNA synthesis in chondrocytes and cause cell cycle arrest, which ultimately leads to cartilage tissue destruction,^42^ and this provides ideas for further exploration of FGF19 promotion of PD/SD. Additionally, FGF19 stimulates osteoblast development and blocks osteoclastogenesis through the Wnt/β‐catenin pathway, which results in increased bone production and proliferation,^43^ which may be important in promoting SCS.

The degree of IVDD and LBP was positively connected with TNF expression.^44^ TNF not only contributes significantly to inflammation in the IVDD immunological milieu^45^ but also can induce healthy NP cells to senescence and exacerbate IVDD.^46^ Our study found that TNF can promote SCS, and TNF‐β can increase the risk of PD/SD. Ossification of the ligamentum flavum is the main cause of spinal stenosis, and TNF not only promotes the proliferation of primary cells of the thoracic ossification of the ligamentum flavum but also enhances osteoblast differentiation to promote SCS onset.^47^ Complementing TNF‐α, TNF‐β can also erode cartilage by creating an inflammatory milieu via the NF‐κB pathway.^48^ There are fewer studies on TNF‐β in IVDD, and it is interesting further to explore the role of TNF‐β in PD/SD.

Studies have shown that estrogen's antiapoptotic actions in IVD can ameliorate spinal degeneration^49^ and also alleviate the degeneration of cartilage endplates, so estrogen deficiency is considered to be an important cause of IVDD and LBP.^50^ SULT1A1, the most important SULT isoform in metabolism and detoxification, can contribute to the sulfation of estrogen, leading to its inactivation.^51^ The study found that SULT1A1 can increase the risk of PD/SD, so the mechanism related to the promotion of the development of SDDs by SULT1A1 through the reduction of estrogen levels is a research direction that deserves attention. One of the characteristics of PD/SD is IVD fibrosis, and there is a significant correlation between the degree of fibrosis and IVDD severity.^52^ The u‐PA/urokinase‐type plasminogen activator receptor (u‐PAR) system, as a fibrinolytic factor, can play an important role in fibrotic diseases by regulating the inflammatory response and immune homeostasis.^53^ In addition, u‐PA can promote angiogenesis and remodel cartilage.^54^ According to our research, PD/SD risk is lowered by genetically predicted u‐PA, which offers genetic support for the clinical injection of u‐PA into IVD tissues as a means of treating SDDs.

### 4.3 Associations of CIPs with spondylolisthesis/spondylolysis

The family of proteases known as deubiquitinases (DUBs) has six subclasses, one of which is JAMM/MPN domain‐associated metallopeptidases (JAMMs). DUBs play an important role in bone remodeling by regulating osteoblasts and osteoclasts, and STAMBP/AMSH is an important subtype in JAMMs.^55^ Studies showed that STAMBP can increase NACHT, LRR and PYD domains‐containing protein 7 (NALP7) inflammasome and IL‐1β levels to promote inflammation through deubiquitination.^56^ On the other hand, we discovered that STAMBP could raise the risk of spondylolisthesis/spondylolysis. Other than causing inflammation, not much is known about the function of STAMBP in spinal diseases and bone homeostasis regulation. The glycoprotein known as CD6, which is mostly expressed on the surface of T cells, has received greater attention in the context of immunological disorders.^57^ CD6 binding to activated leukocyte cell adhesion molecule (ALCAM) is the most classical signaling pathway,^58^ and ALCAM+ perichondrial cells can participate in vascular invasion by recruiting osteoclasts,^59^ so CD6 may be associated with osteoclast viability. CD6 transcripts generate multiple CD6 isoforms by alternative splicing. However, there are few reports related to the involvement of CD6/CD6 isoform in bone homeostasis as well as SDDs, and we found that genetically predicted CD6 isoform promotes the onset of spondylolisthesis/spondylolysis. The primary function of MCP2/C‐C motif chemokine ligand 8 (CCL8) is to act as a chemoattractant to induce migration of associated cells. MCP2 can regulate inflammation, ameliorate fibrosis, and promote fracture healing by inducing the migration of osteoprogenitor cells.^60^, ^61^ We discovered that MCP2 can lower the likelihood of spondylolisthesis/spondylolysis, and more research is necessary to determine its involvement in spondylolysis and other nonunions. Among these 91 CIPs, TGF‐β1 was not found. However, LAP‐TGF‐β1, a TGF‐β1 precursor protein, can split into LAP and TGF‐β1.^62^ TGF‐β1 is secreted by osteoblasts and can promote the differentiation of preosteoblasts and inhibit osteoclast proliferation, and it plays an important role in bone reconstruction as the most abundant growth factor in human bone.^63^ Clinical studies have reported that TGF‐β1 levels are significantly lower in patients with osteoporosis and that TGF‐β1 levels are positively correlated with BMD of the spine.^64^ Because it promotes osteoblast viability and prevents apoptosis, the TGF‐β1 pathway is very significant in osteoporosis.^65^ In addition, TGF‐β1 can promote the healing of fractures,^66^ the repair of bone defects,^67^ the regeneration of nonunion,^68^ and the recovery of bone regeneration disability^69^ through a variety of mechanisms. It was discovered that LAP‐TGF‐β1 can lower the risk of spondylolisthesis/spondylolysis.

This study has several strengths, as follows. First, this is the first MR analysis that we are aware of that investigates the causal associations between 91 CIPs and 4 SDDs. Second, A bidirectional TSMR analysis eliminates the effects of reverse causality and lessens the bias brought about by confounding and environmental factors. Third, Numerous studies have been reported on the relationships between CIPs and SDDs, but there are conflicting, and the MR analysis based on gene prediction in this study can provide genetic evidence for research in this area. Certainly, this study has some limitations. First, MR analysis provides evidence of the causal direction of the genetic association between exposure and outcome, but needs to be further validated by rigorous RCTs. Second, although the sensitivity analyses of MR indicate the validity and robustness of the results, there is still the possibility of residual heterogeneity. Finally, we only used GWAS data from European ancestry for our MR analysis, so results should be interpreted cautiously when extrapolated to other populations.

## 5 CONCLUSION

The development of inflammation is one of the main pathogenic characteristics of SDDs, and using MR analysis, we demonstrated causal relationships between several genetically predicted CIPs and four different forms of SDDs (CS, PD/SD, SCS, and spondylolisthesis/spondylolysis). This MR analysis effectively reduces the bias caused by confounding factors, environmental factors, and reverse causality, and the results have good validity and robustness. Our study offers solid genetic support for a thorough investigation into the role of CIPs in the pathogenic mechanism of SDDs and offers insightful information about the hunt for potential therapeutic targets for SDDs.

## AUTHOR CONTRIBUTIONS

QZ, RL, and DW consulted the literature and prepared materials. QZ, RL, and DW analyzed the data. QZ, RL, and DW: drawn up the manuscript. WX, and CZ devised the concept and supervised the study. All authors contributed to the article and approved the submitted version.

## FUNDING INFORMATION

The Natural Science Foundation of Fujian Province (No. 2021 J02035), National Natural Science Foundation of China (No. 82072263) and Fujian Provincial Health Technology Project (No. 2020CXA039) supported this study.

## CONFLICT OF INTEREST STATEMENT

The authors declare no conflict of interest.

## Supporting information


**Data S1.** Supporting information.

## Data Availability

The original data associated with this study are available within the paper and its additional files.
