# Supplementary material for: Effects of circulating inflammatory proteins on spinal degenerative diseases: Evidence from genetic correlations and Mendelian randomization study
Source: JOR Spine. 2024 Jun 17;7(2):e1346. doi: 10.1002/jsp2.1346 (PMC11183170; doi:10.1002/jsp2.1346)
Supplement: Supplementary file 1 — Data S1. Supporting information. [file JSP2-7-e1346-s001.zip › Supplementary figure.pdf]

Supplementary Material

Figure S1. Assumptions in MR analysis. 1
Figure S2. Scatter plots of causal associations between exposures (CIPs) and outcome (CS).. 2
Figure S3. Scatter plots of causal associations between exposures (CIPs) and outcome (PD/SD). 3
Figure S4. Scatter plots of causal associations between exposures (CIPs) and outcome (SCS). 4
Figure S5. Scatter plots of causal associations between exposures (CIPs) and outcome (spondylolisthesis/spondylolysis).. 5
Figure S6. Forest plots of causal associations between exposures (CIPs) and outcome (CS) 6
Figure S7. Forest plots of causal associations between exposures (CIPs) and outcome (PD/SD). . 7
Figure S8. Forest plots of causal associations between exposures (CIPs) and outcome (SCS). 8
Figure S9. Forest plots of causal associations between exposures (CIPs) and outcome (spondylolisthesis/spondylolysis). . 9
Figure S10. Funnel plots of causal associations between exposures (CIPs) and outcome (CS) 10
Figure S11. Funnel plots of causal associations between exposures (CIPs) and outcome (PD/SD). 11
Figure S12. Funnel plots of causal associations between exposures (CIPs) and outcome (SCS). 12
Figure S13. Funnel plots of causal associations between exposures (CIPs) and outcome (spondylolisthesis/spondylolysis). . 13
Figure S14. Leave-on-out plots of causal associations between exposures (CIPs) and outcome (CS) 14
Figure S15. Leave-on-out plots of causal associations between exposures (CIPs) and outcome (PD/SD) 15
Figure S16. Leave-on-out plots of causal associations between exposures (CIPs) and outcome (SCS) 16
Figure S17. Leave-on-out plots of causal associations between exposures (CIPs) and outcome (spondylolisthesis/spondylolysis). 17
Figure S18. Scatter plots of causal associations between exposure (CS) and outcomes (CIPs). 18
Figure S19. Scatter plots of causal associations between exposure (PD/SD) and outcomes (CIPs). 19
Figure S20. Scatter plots of causal associations between exposure (SCS) and outcomes (CIPs). 20
Figure S21. Scatter plots of causal associations between exposure (spondylolisthesis/spondylolysis) and outcomes (CIPs). 21
Figure S22. Forest plots of causal associations between exposure (CS) and outcomes (CIPs). 22
Figure S23. Forest plots of causal associations between exposure (PD/SD) and outcomes (CIPs). 23

Figure S24. Forest plots of causal associations between exposure (SCS) and outcomes (CIPs). 24
Figure S25. Forest plots of causal associations between exposure (spondylolisthesis/spondylolysis) and outcomes (CIPs). 25
Figure S26. Funnel plots of causal associations between exposure (CS) and outcomes (CIPs). 26
Figure S27. Funnel plots of causal associations between exposure (PD/SD) and outcomes (CIPs). 27
Figure S28. Funnel plots of causal associations between exposure (SCS) and outcomes (CIPs). 28
Figure S29. Funnel plots of causal associations between exposure (spondylolisthesis/spondylolysis) and outcomes (CIPs). 29
Figure S30. Leave-on-out plots of causal associations between exposure (CS) and outcomes (CIPs). 30
Figure S31. Leave-on-out plots of causal associations between exposure (PD/SD) and outcomes (CIPs). 31
Figure S32. Leave-on-out plots of causal associations between exposure (SCS) and outcomes (CIPs). 32
Figure S33. Leave-on-out plots of causal associations between exposure (spondylolisthesis/spondylolysis) and outcomes (CIPs). 33

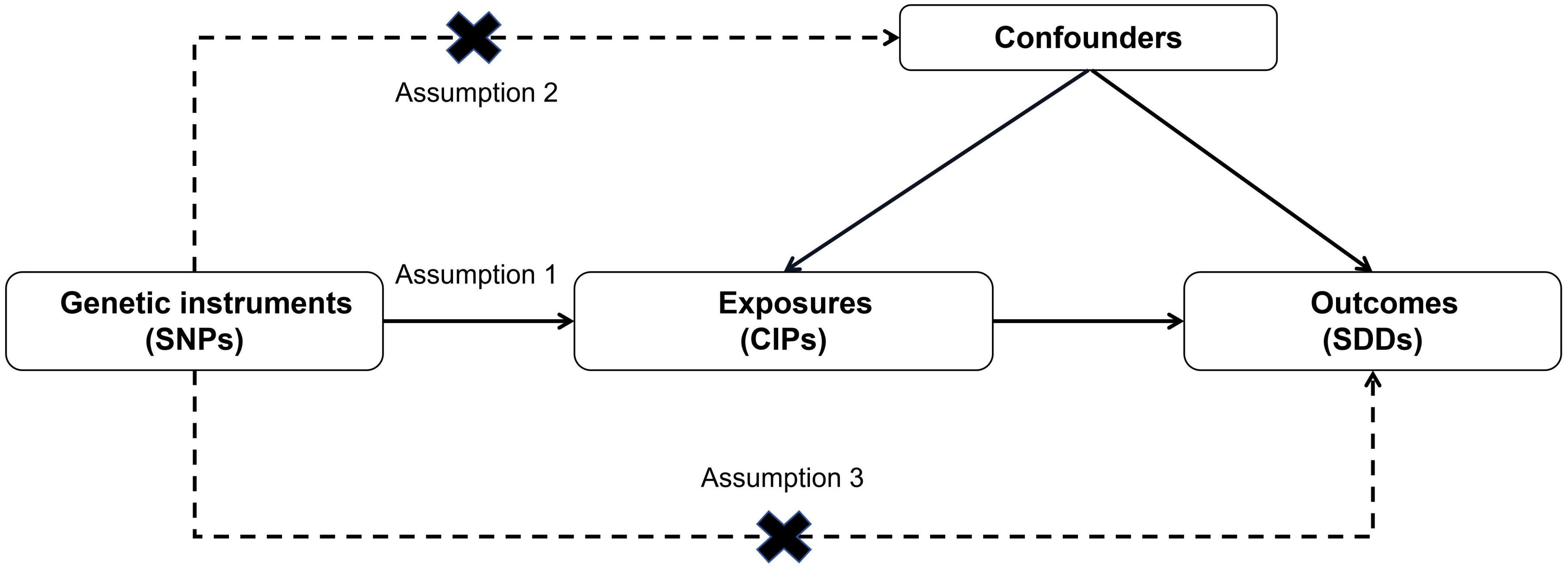

Figure S1. Assumptions in MR analysis. Three assumptions of MR are as follows: (1) Assumption 1: Genetic instruments must be significantly associated with the exposure; (2) Assumption 2: Genetic instruments are independent of confounders; (3) Assumption 3: Genetic instruments affect the outcome only via exposure.

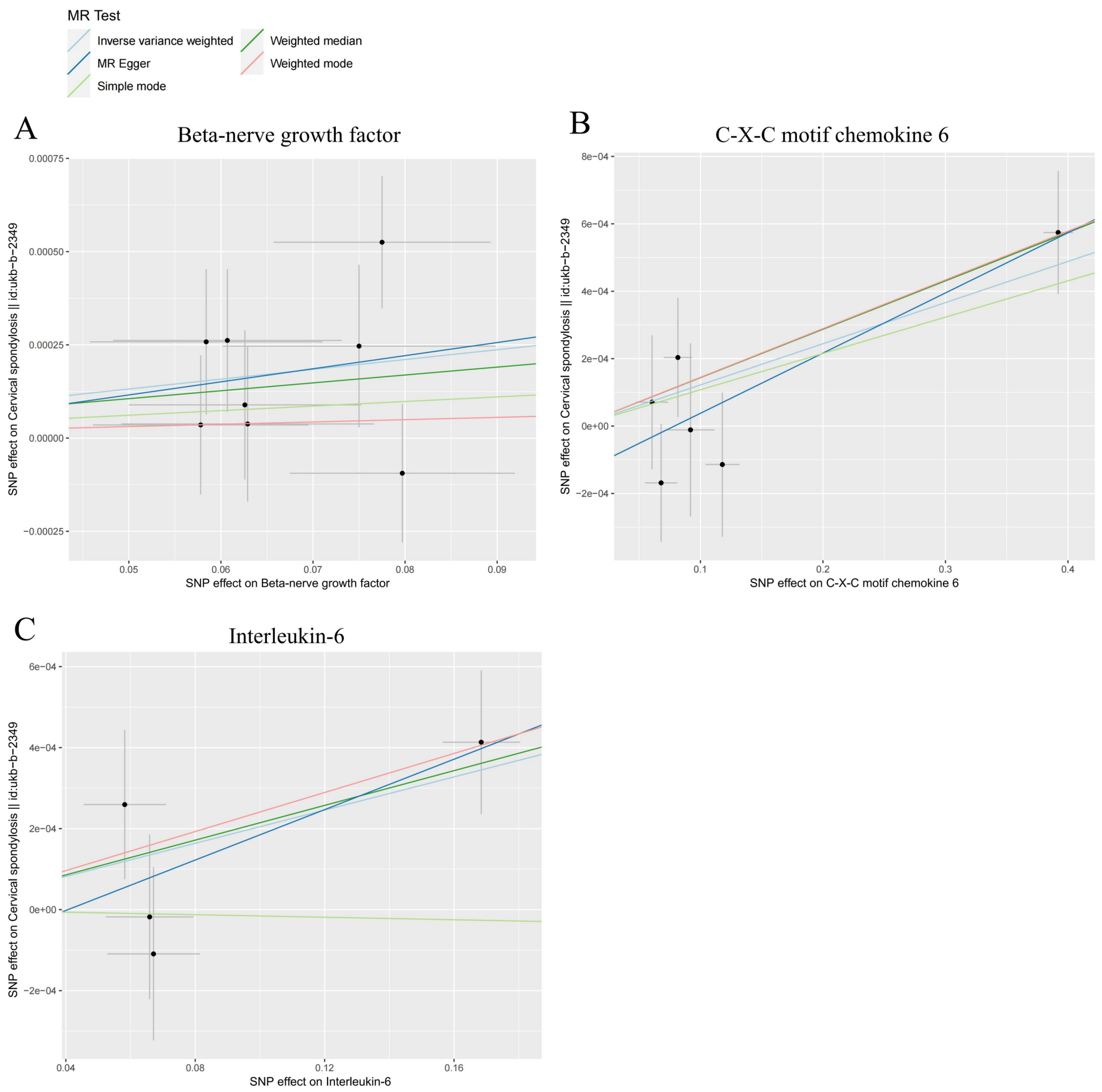

Figure S2. Scatter plots of causal associations between exposures (CIPs) and outcome (CS). (A) Scatter plot between beta-nerve growth factor and CS; (B) Scatter plot between C-X-C motif chemokine 6 and CS; (C) Scatter plot between interleukin-6 and CS.

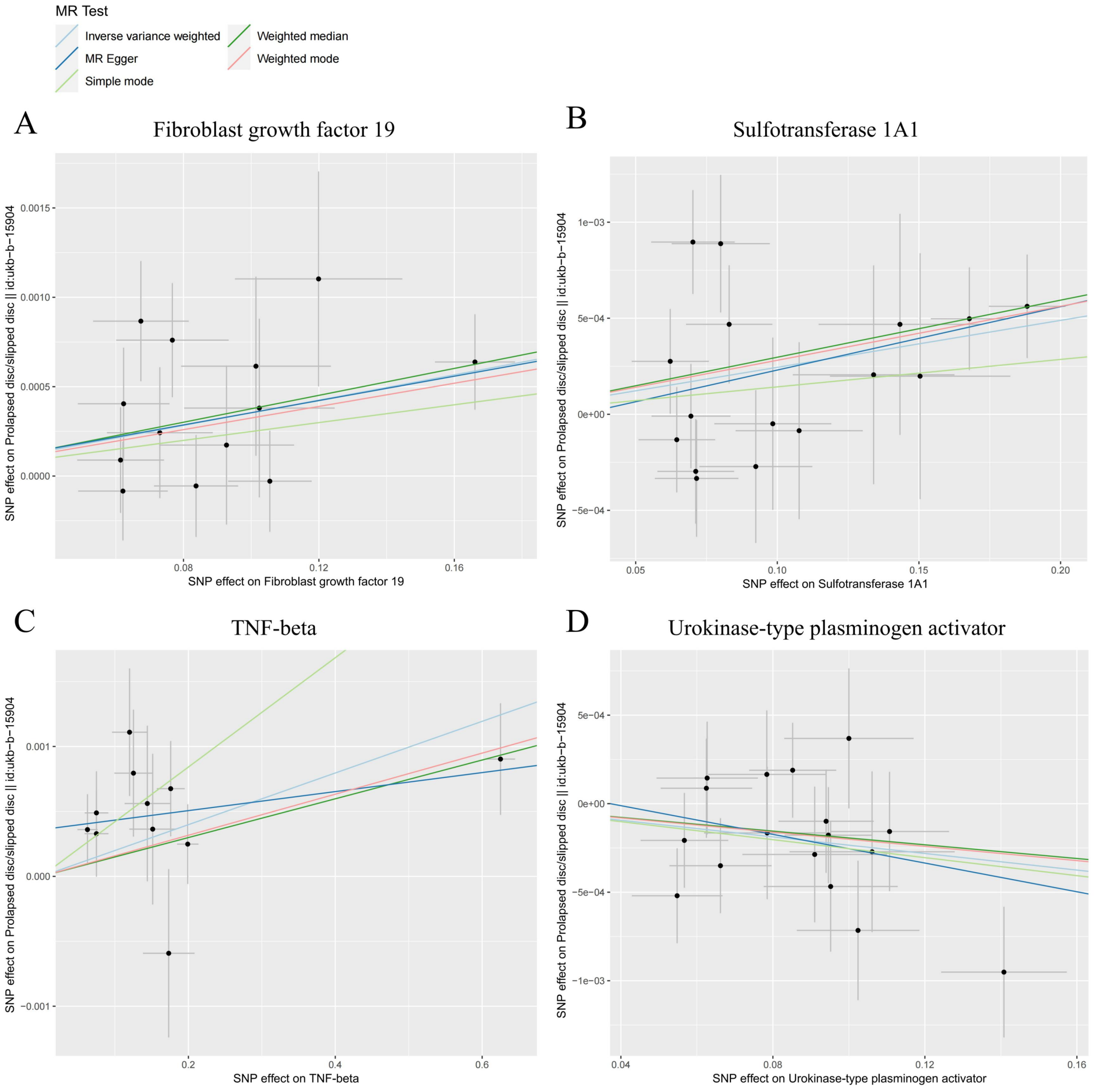

Figure S3. Scatter plots of causal associations between exposures (CIPs) and outcome (PD/SD). (A) Scatter plot between fibroblast growth factor 19 and PD/SD; (B) Scatter plot between sulfotransferase 1A1 and PD/SD; (C) Scatter plot between TNF-beta and PD/SD; (D) Scatter plot between urokinase-type plasminogen activator and PD/SD.

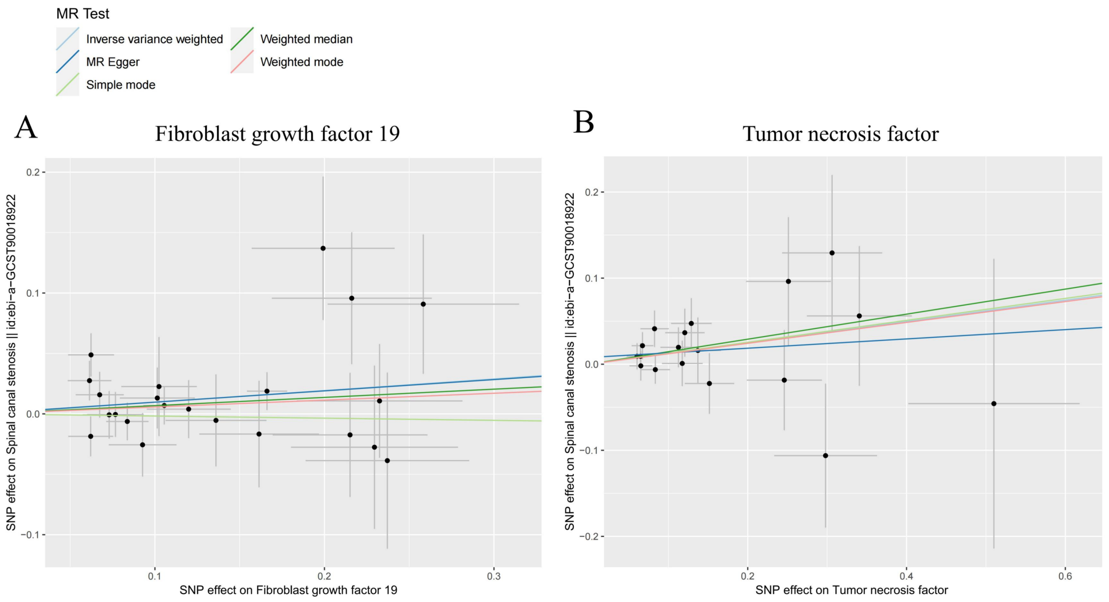

Figure S4. Scatter plots of causal associations between exposures (CIPs) and outcome (SCS). (A) Scatter plot between fibroblast growth factor 19 and SCS; (B) Scatter plot between tumor necrosis factor and SCS.

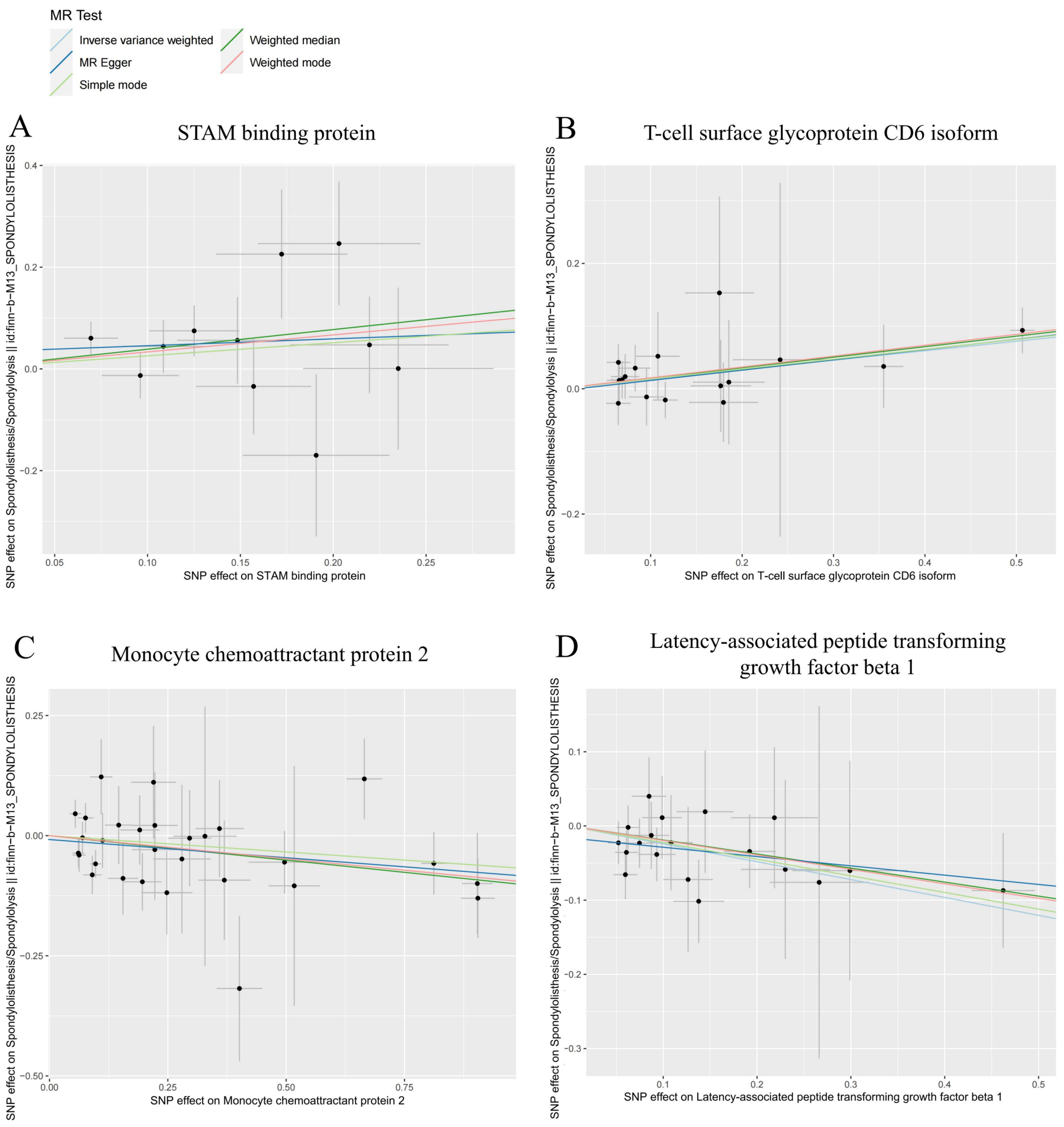

Figure S5. Scatter plots of causal associations between exposures (CIPs) and outcome (spondylolisthesis/spondylolysis). (A) Scatter plot between STAM binding protein and spondylolisthesis/spondylolysis; (B) Scatter plot between T-cell surface glycoprotein CD6 isoform and spondylolisthesis/spondylolysis; (C) Scatter plot between monocyte chemoattractant protein 2 and spondylolisthesis/spondylolysis; (D) Scatter plot between latency-associated peptide transforming growth factor beta 1 and spondylolisthesis/spondylolysis.

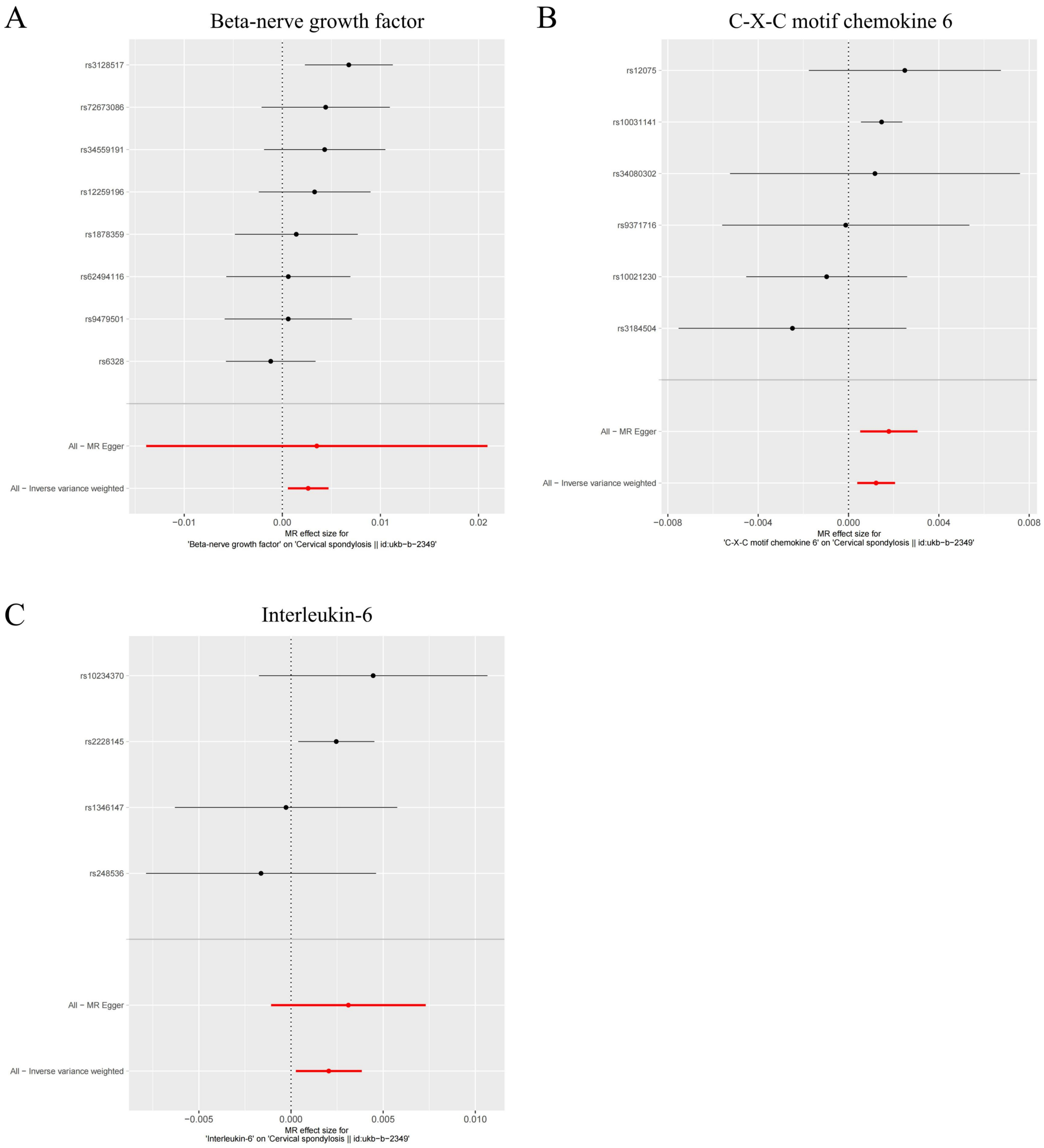

Figure S6. Forest plots of causal associations between exposures (CIPs) and outcome (CS). (A) Forest plot between beta-nerve growth factor and CS; (B) Forest plot between C-X-C motif chemokine 6 and CS; (C) Forest plot between interleukin-6 and CS.

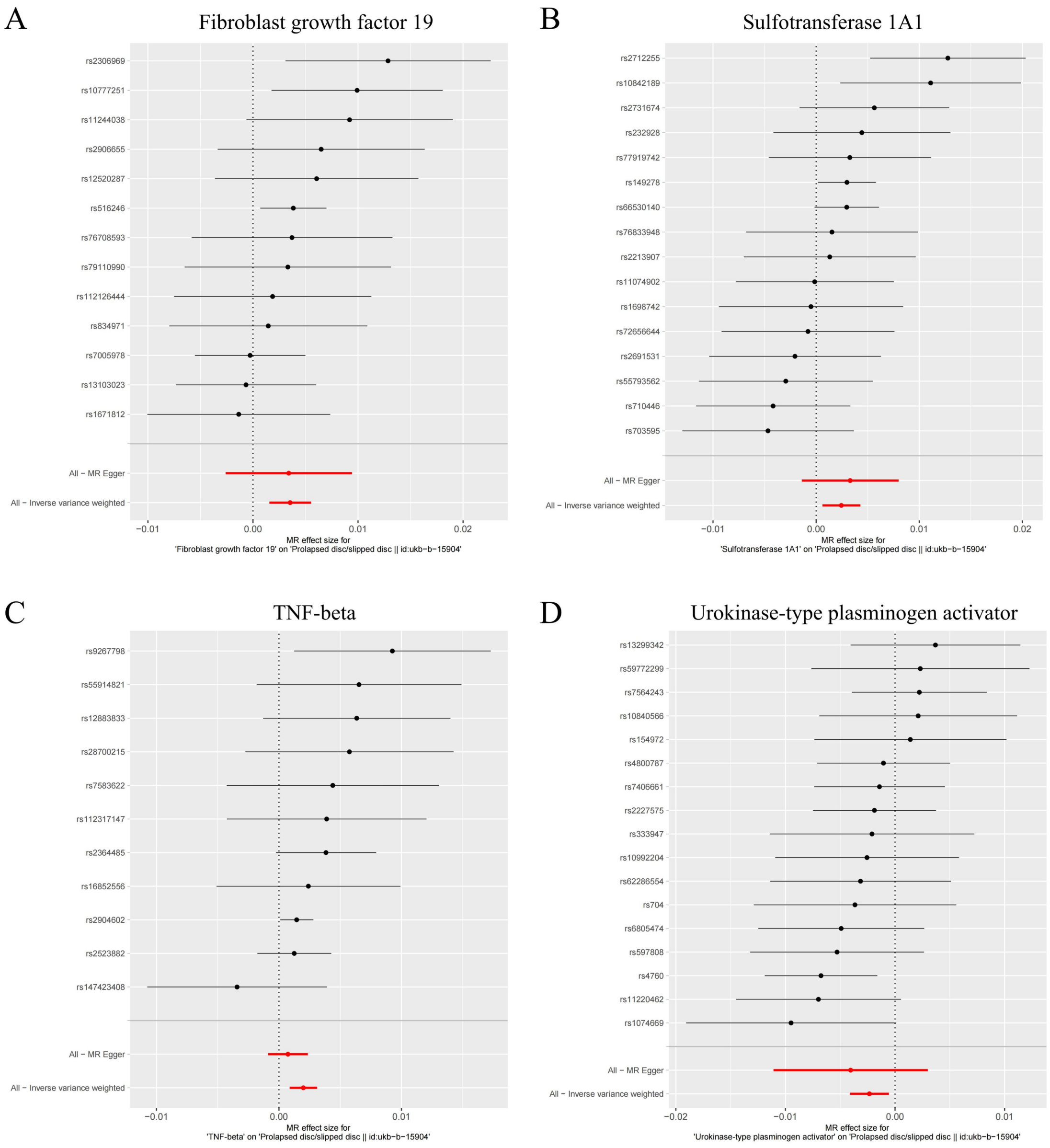

Figure S7. Forest plots of causal associations between exposures (CIPs) and outcome (PD/SD). (A) Forest plot between fibroblast growth factor 19 and PD/SD; (B) Forest plot between sulfotransferase 1A1 and PD/SD; (C) Forest plot between TNF-beta and PD/SD; (D) Forest plot between urokinase-type plasminogen activator and PD/SD.

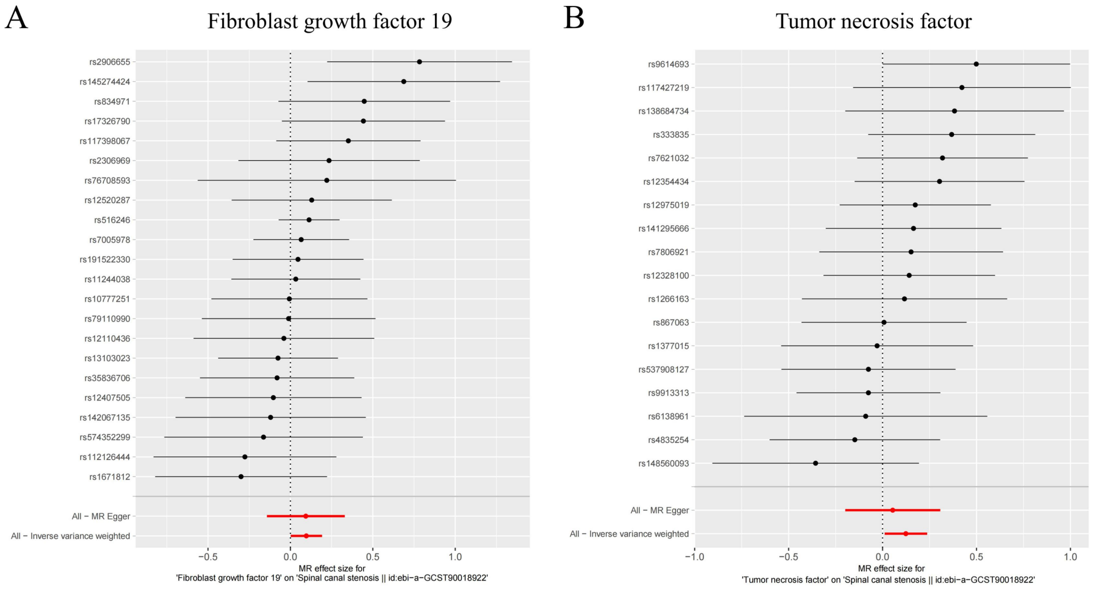

Figure S8. Forest plots of causal associations between exposures (CIPs) and outcome (SCS). (A) Forest plot between fibroblast growth factor 19 and SCS; (B) Forest plot between tumor necrosis factor and SCS.

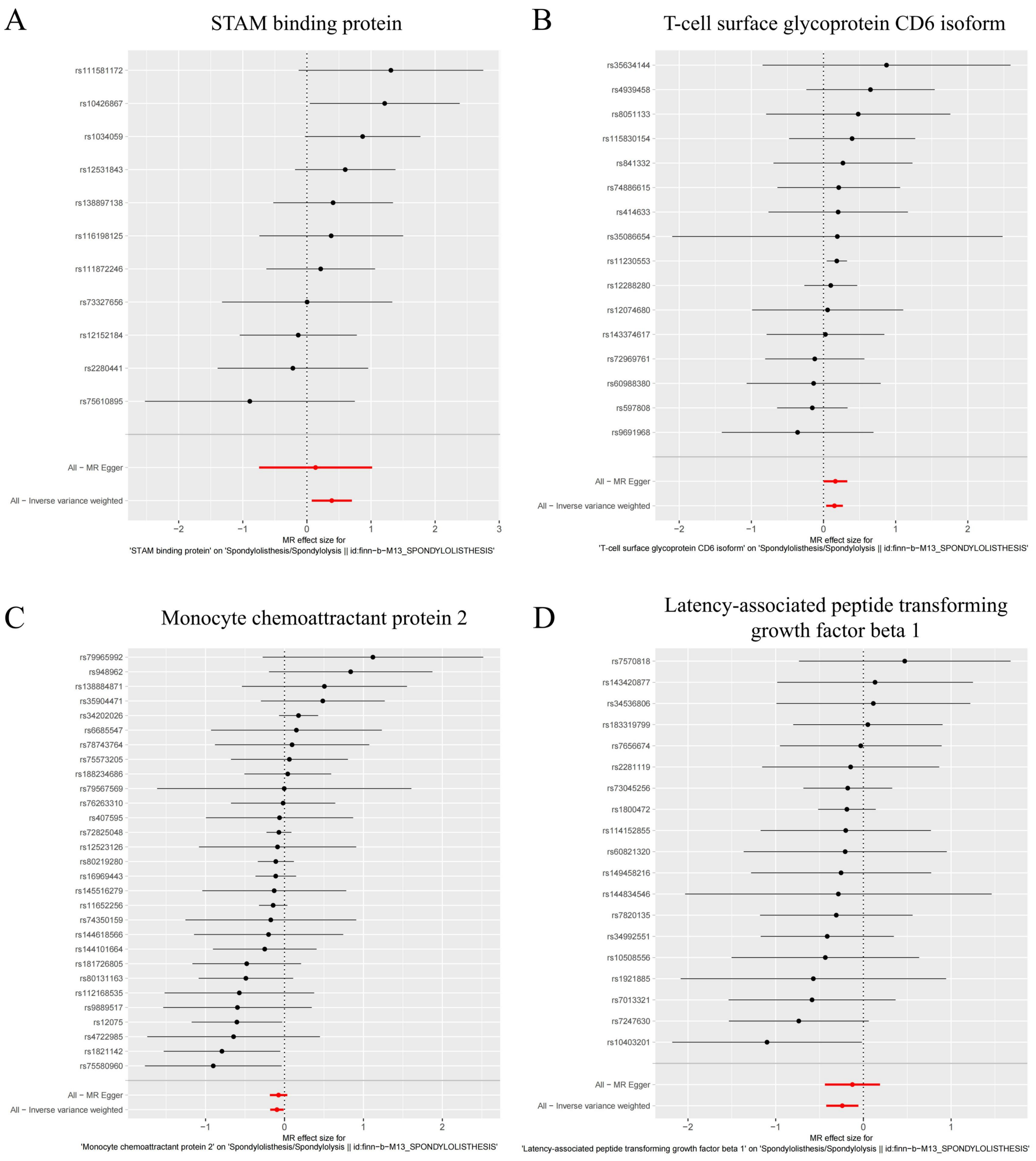

Figure S9. Forest plots of causal associations between exposures (CIPs) and outcome (spondylolisthesis/spondylolysis). (A) Forest plot between STAM binding protein and spondylolisthesis/spondylolysis; (B) Forest plot between T-cell surface glycoprotein CD6 isoform and spondylolisthesis/spondylolysis; (C) Forest plot between monocyte chemoattractant protein 2 and spondylolisthesis/spondylolysis; (D) Forest plot between latency-associated peptide transforming growth factor beta 1 and spondylolisthesis/spondylolysis.

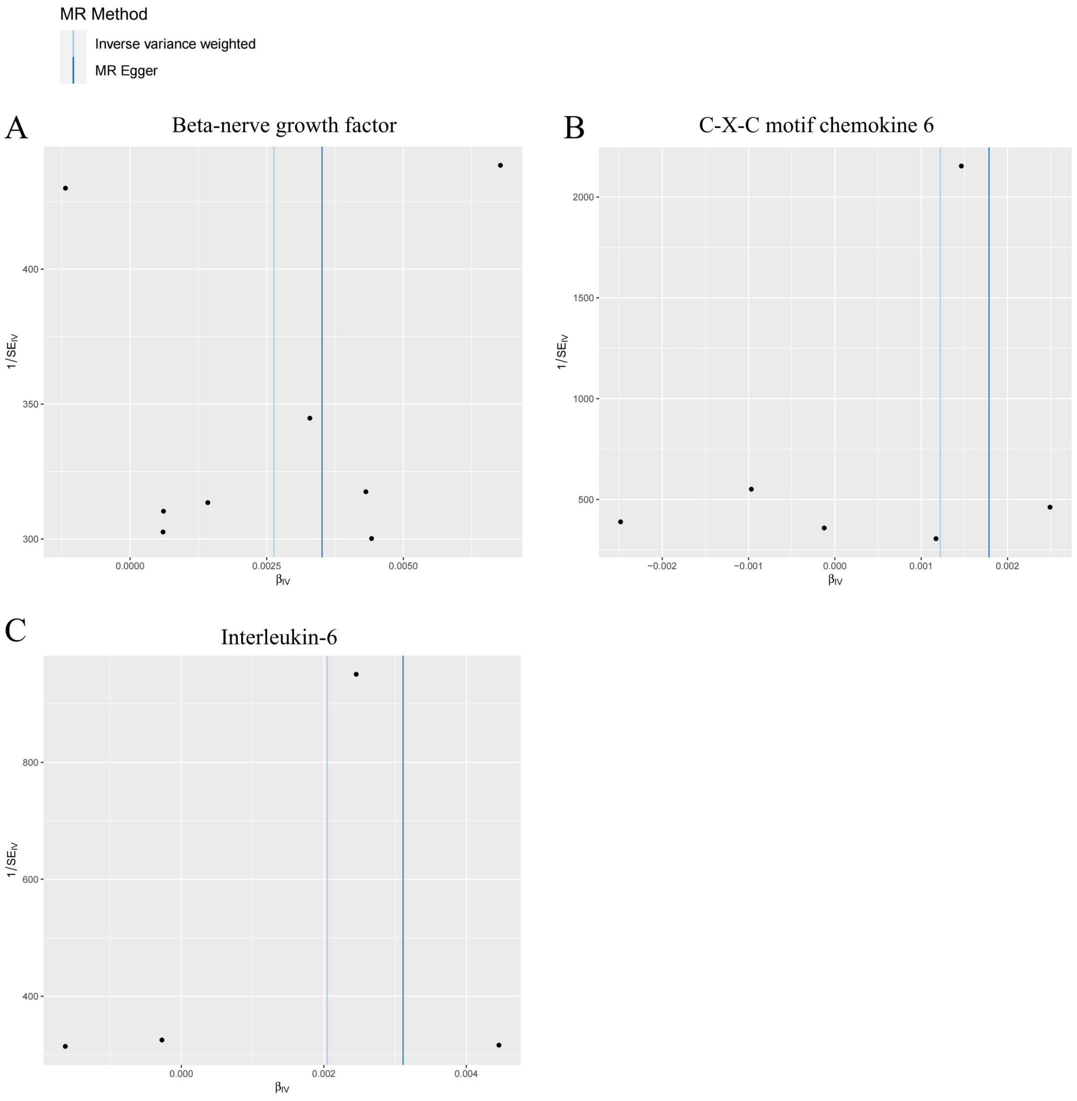

Figure S10. Funnel plots of causal associations between exposures (CIPs) and outcome (CS). (A) Funnel plot between beta-nerve growth factor and CS; (B) Funnel plot between C-X-C motif chemokine 6 and CS; (C) Funnel plot between interleukin-6 and CS.

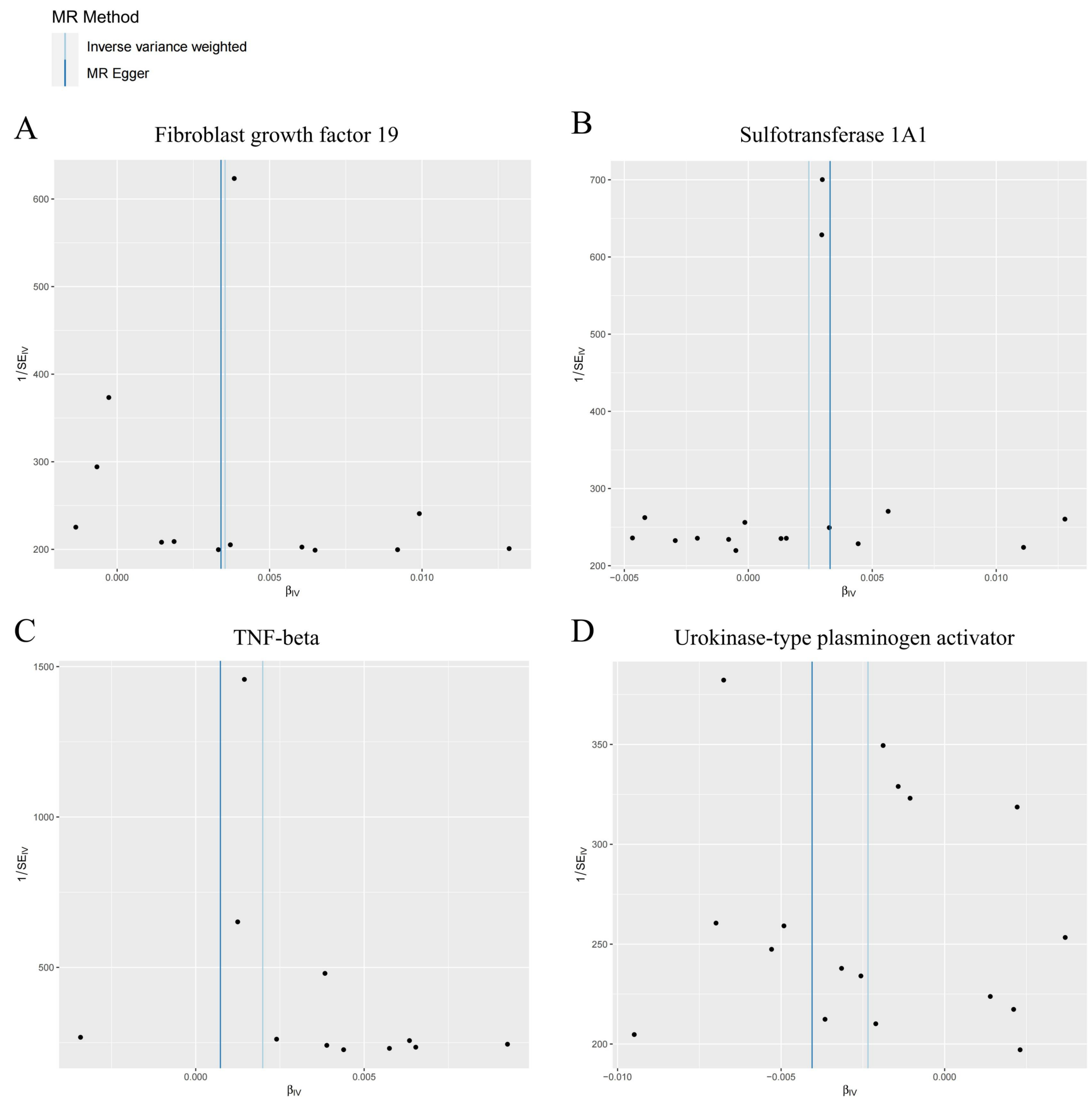

Figure S11. Funnel plots of causal associations between exposures (CIPs) and outcome (PD/SD). (A) Funnel plot between fibroblast growth factor 19 and PD/SD; (B) Funnel plot between sulfotransferase 1A1 and PD/SD; (C) Funnel plot between TNF-beta and PD/SD; (D) Funnel plot between urokinase-type plasminogen activator and PD/SD.

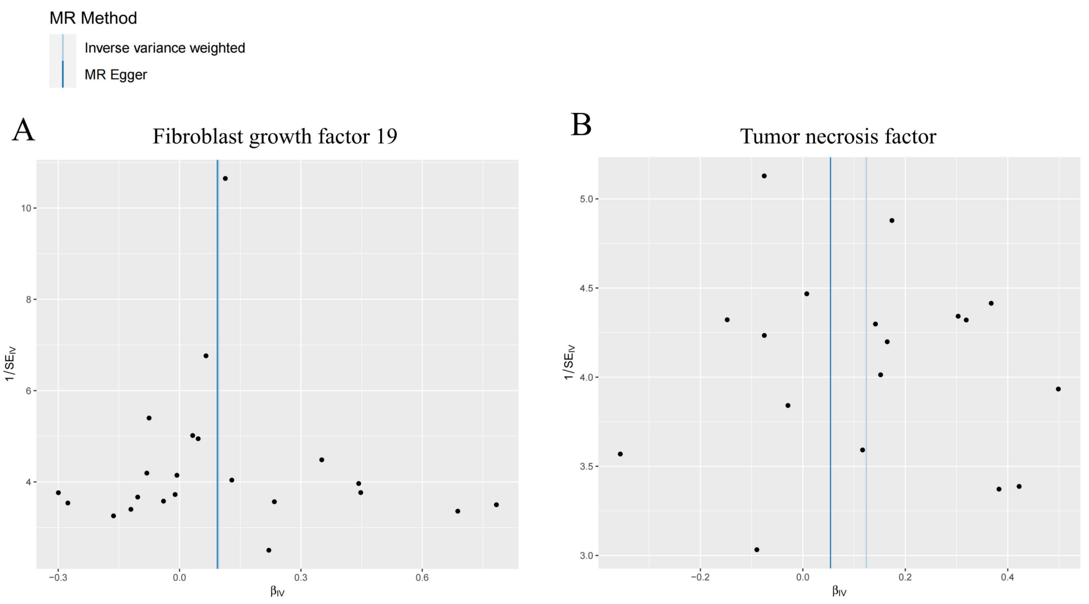

Figure S12. Funnel plots of causal associations between exposures (CIPs) and outcome (SCS). (A) Funnel plot between fibroblast growth factor 19 and SCS; (B) Funnel plot between tumor necrosis factor and SCS.

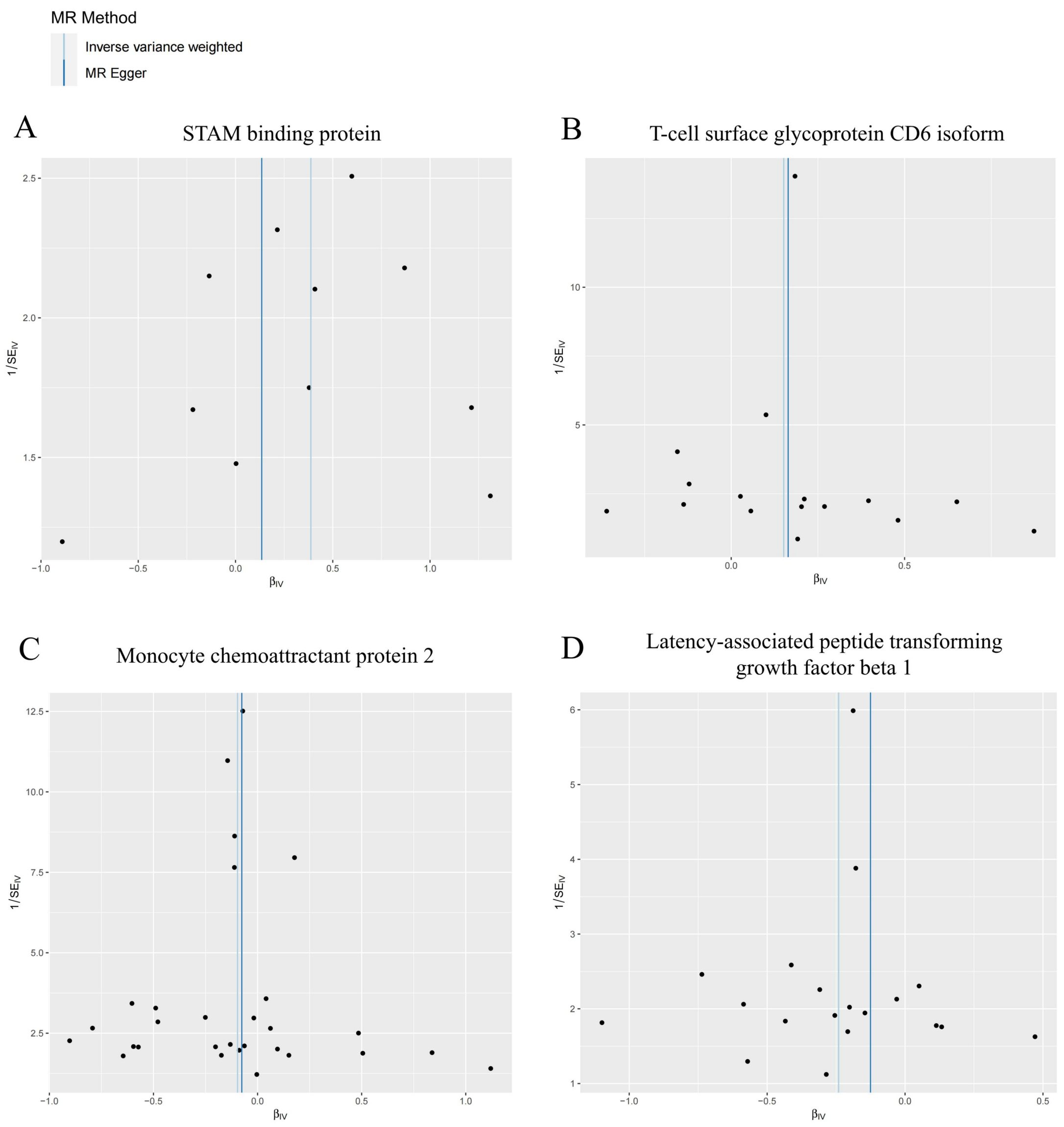

Figure S13. Funnel plots of causal associations between exposures (CIPs) and outcome (spondylolisthesis/spondylolysis). (A) Funnel plot between STAM binding protein and spondylolisthesis/spondylolysis; (B) Funnel plot between T-cell surface glycoprotein CD6 isoform and spondylolisthesis/spondylolysis; (C) Funnel plot between monocyte chemoattractant protein 2 and spondylolisthesis/spondylolysis; (D) Funnel plot between latency-associated peptide transforming growth factor beta 1 and spondylolisthesis/spondylolysis.

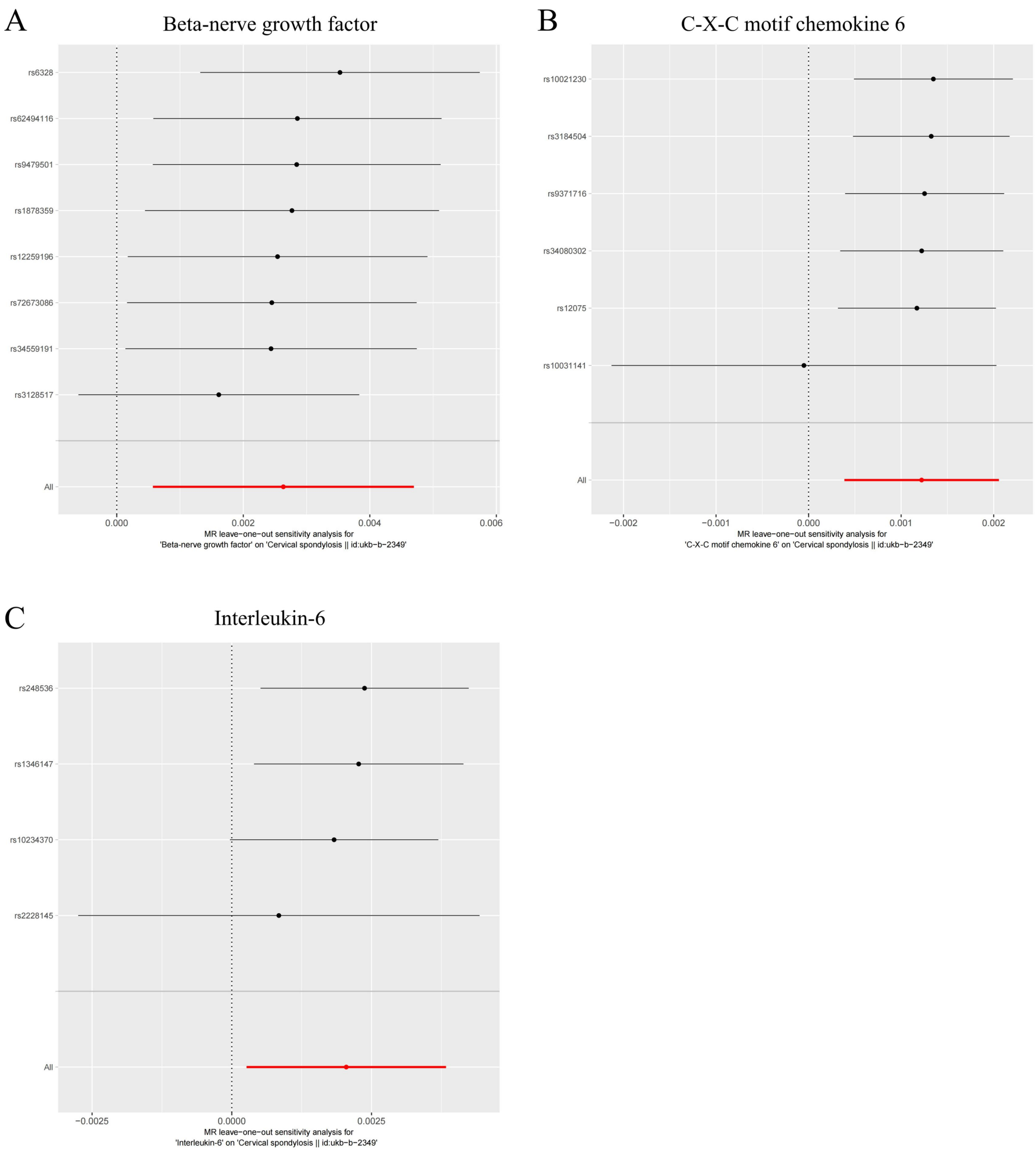

Figure S14. Leave-on-out plots of causal associations between exposures (CIPs) and outcome (CS). (A) Leave-on-out plot between beta-nerve growth factor and CS; (B) Leave-on-out plot between C-X-C motif chemokine 6 and CS; (C) Leave-on-out plot between interleukin-6 and CS.

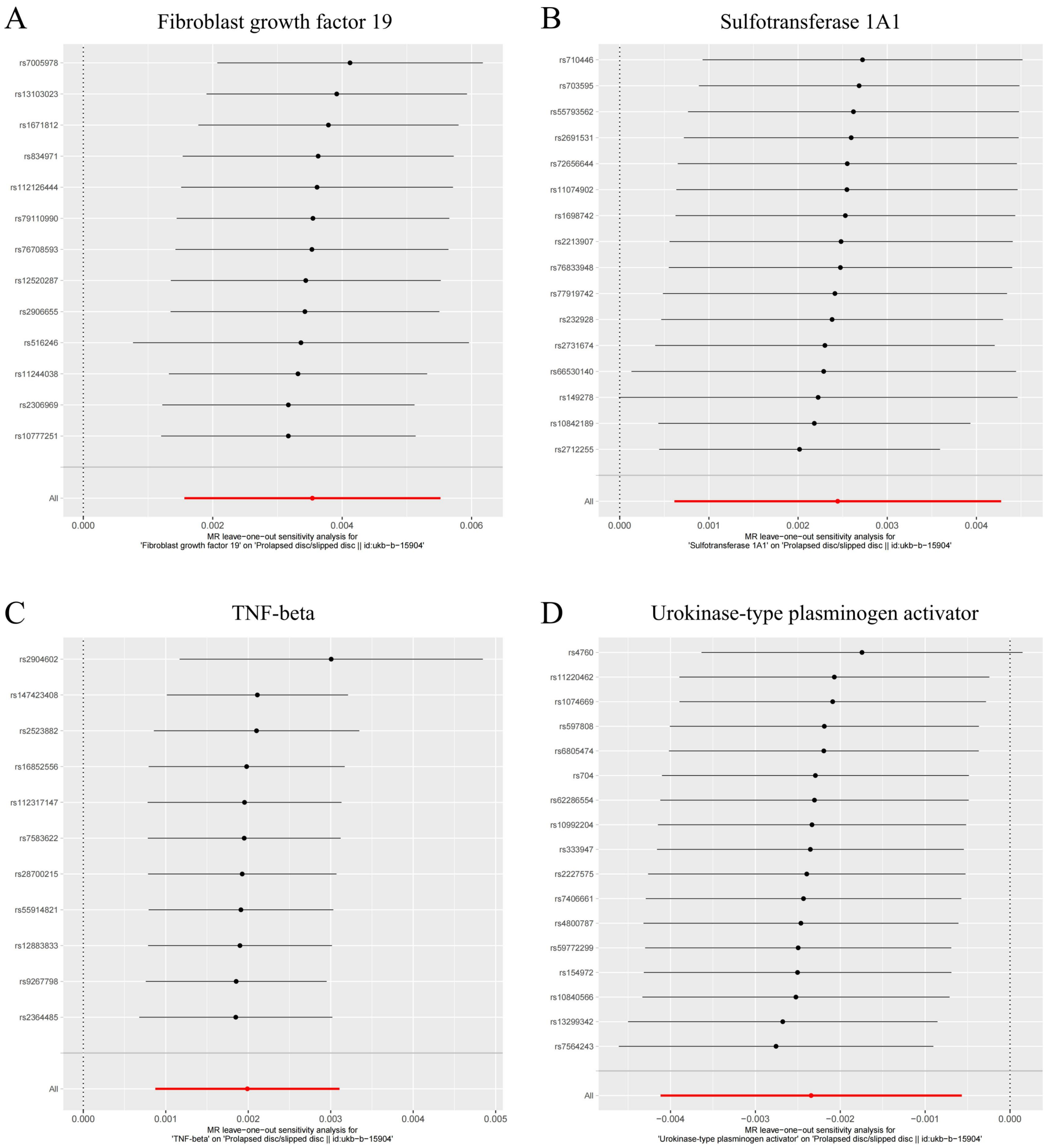

Figure S15. Leave-on-out plots of causal associations between exposures (CIPs) and outcome (PD/SD). (A) Leave-on-out plot between fibroblast growth factor 19 and PD/SD; (B) Leave-on-out plot between sulfotransferase 1A1 and PD/SD; (C) Leave-on-out plot between TNF-beta and PD/SD; (D) Leave-on-out plot between urokinase-type plasminogen activator and PD/SD.

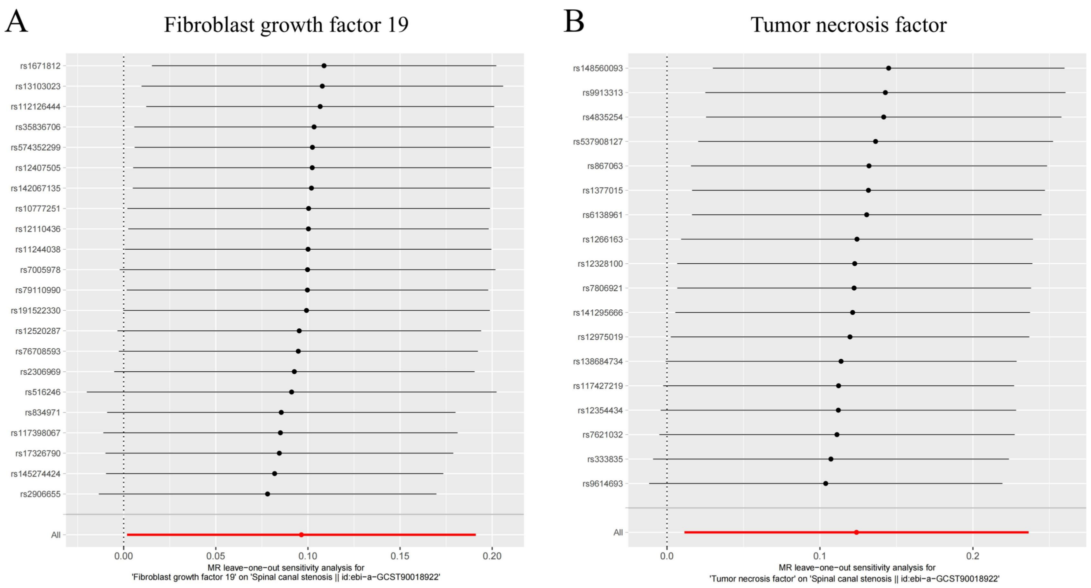

Figure S16. Leave-on-out plots of causal associations between exposures (CIPs) and outcome (SCS). (A) Leave-on-out plot between fibroblast growth factor 19 and SCS; (B) Leave-on-out plot between tumor necrosis factor and SCS.

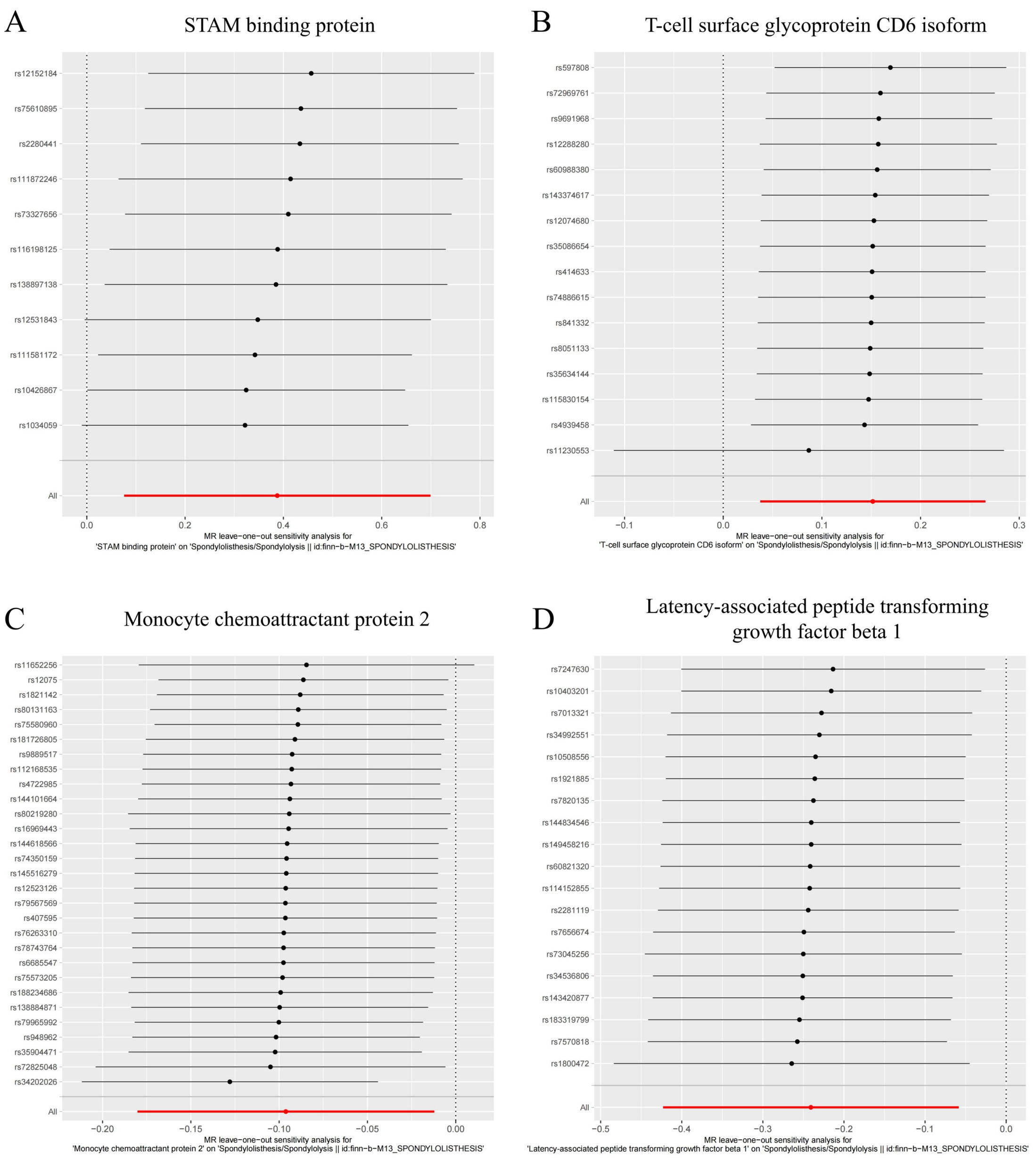

Figure S17. Leave-on-out plots of causal associations between exposures (CIPs) and outcome (spondylolisthesis/spondylolysis). (A) Leave-on-out plot between STAM binding protein and spondylolisthesis/spondylolysis; (B) Leave-on-out plot between T-cell surface glycoprotein CD6 isoform and spondylolisthesis/spondylolysis; (C) Leave-on-out plot between monocyte chemoattractant protein 2 and spondylolisthesis/spondylolysis; (D) Leave-on-out plot between latency-associated peptide transforming growth factor beta 1 and spondylolisthesis/spondylolysis.

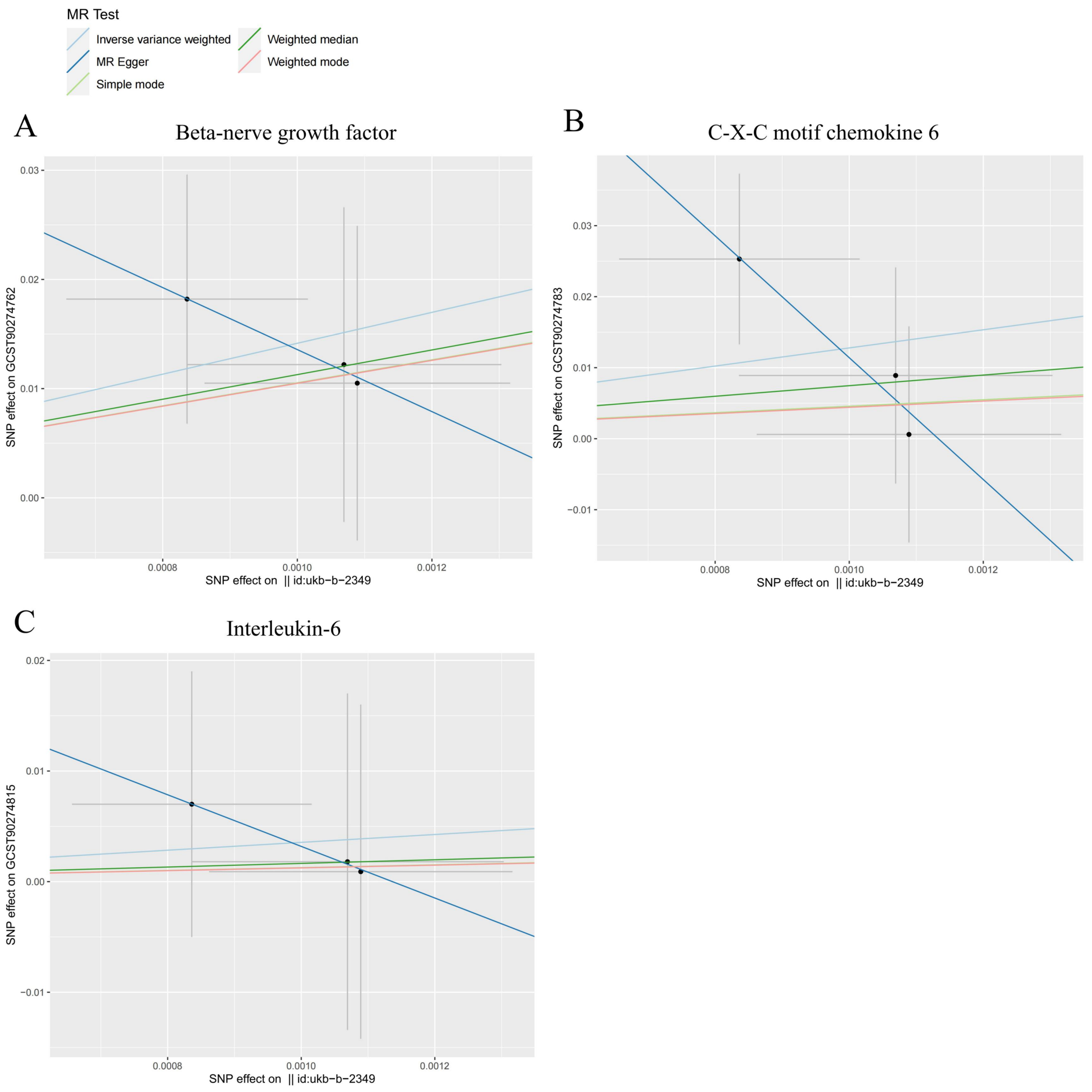

Figure S18. Scatter plots of causal associations between exposure (CS) and outcomes (CIPs). (A) Scatter plot between CS and beta-nerve growth factor; (B) Scatter plot between CS and C-X-C motif chemokine 6; (C) Scatter plot between CS and interleukin-6.

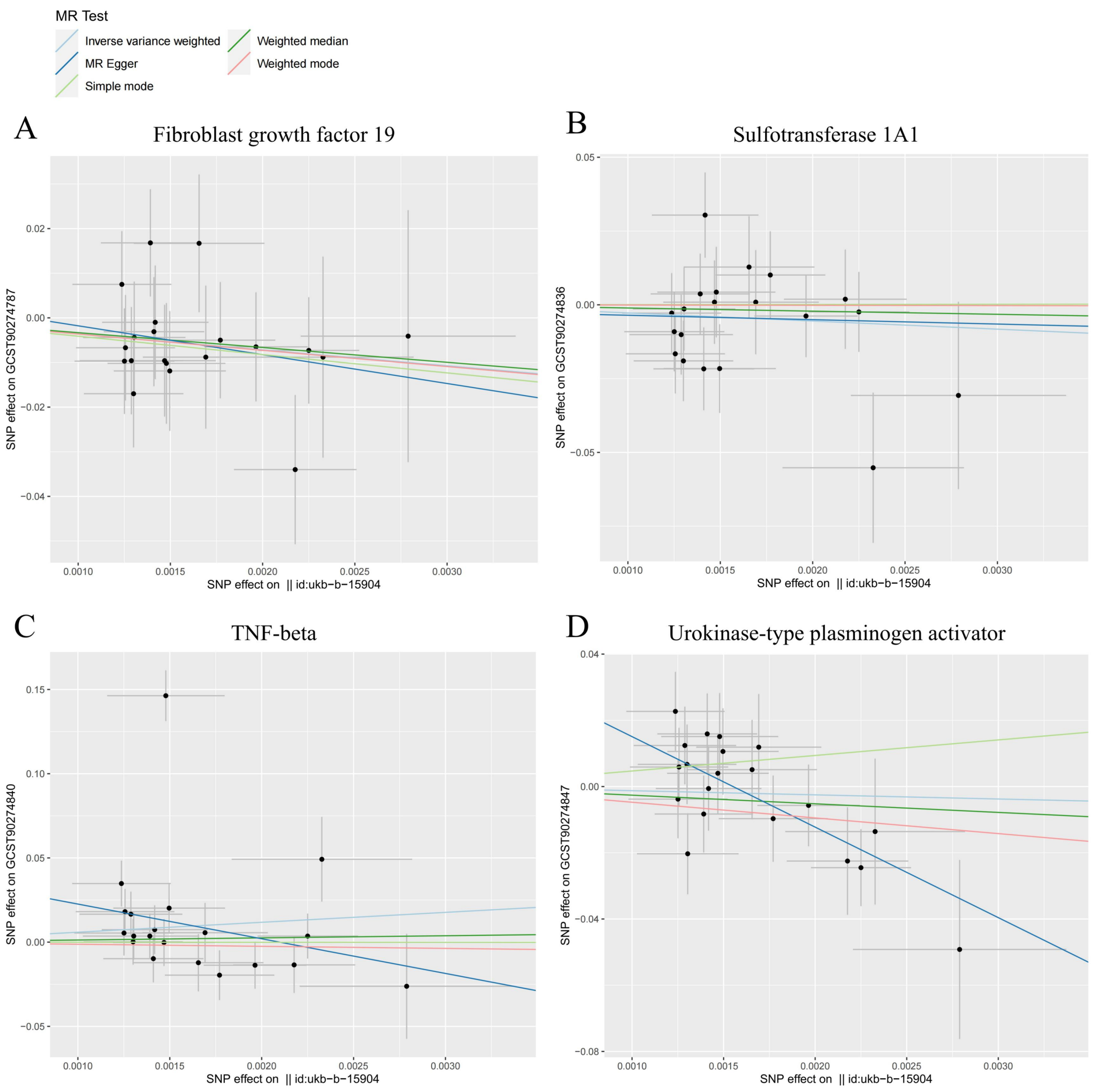

Figure S19. Scatter plots of causal associations between exposure (PD/SD) and outcomes (CIPs). (A) Scatter plot between PD/SD and fibroblast growth factor 19; (B) Scatter plot between PD/SD and sulfotransferase 1A1; (C) Scatter plot between PD/SD and TNF-beta; (D) Scatter plot between PD/SD and urokinase-type plasminogen activator.

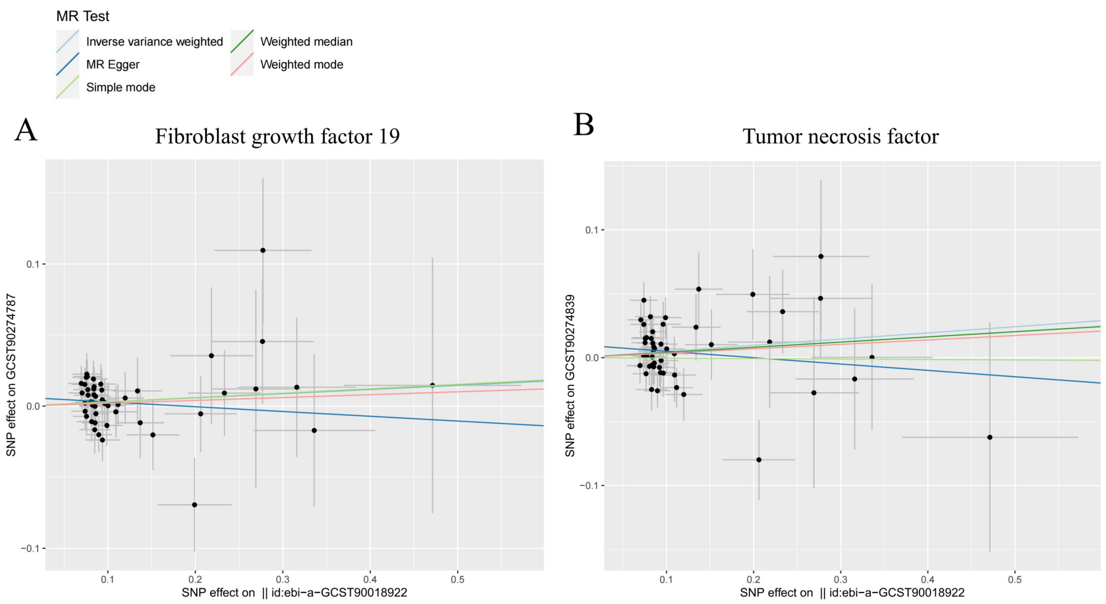

Figure S20. Scatter plots of causal associations between exposure (SCS) and outcomes (CIPs). (A) Scatter plot between SCS and fibroblast growth factor 19; (B) Scatter plot between SCS and tumor necrosis factor.

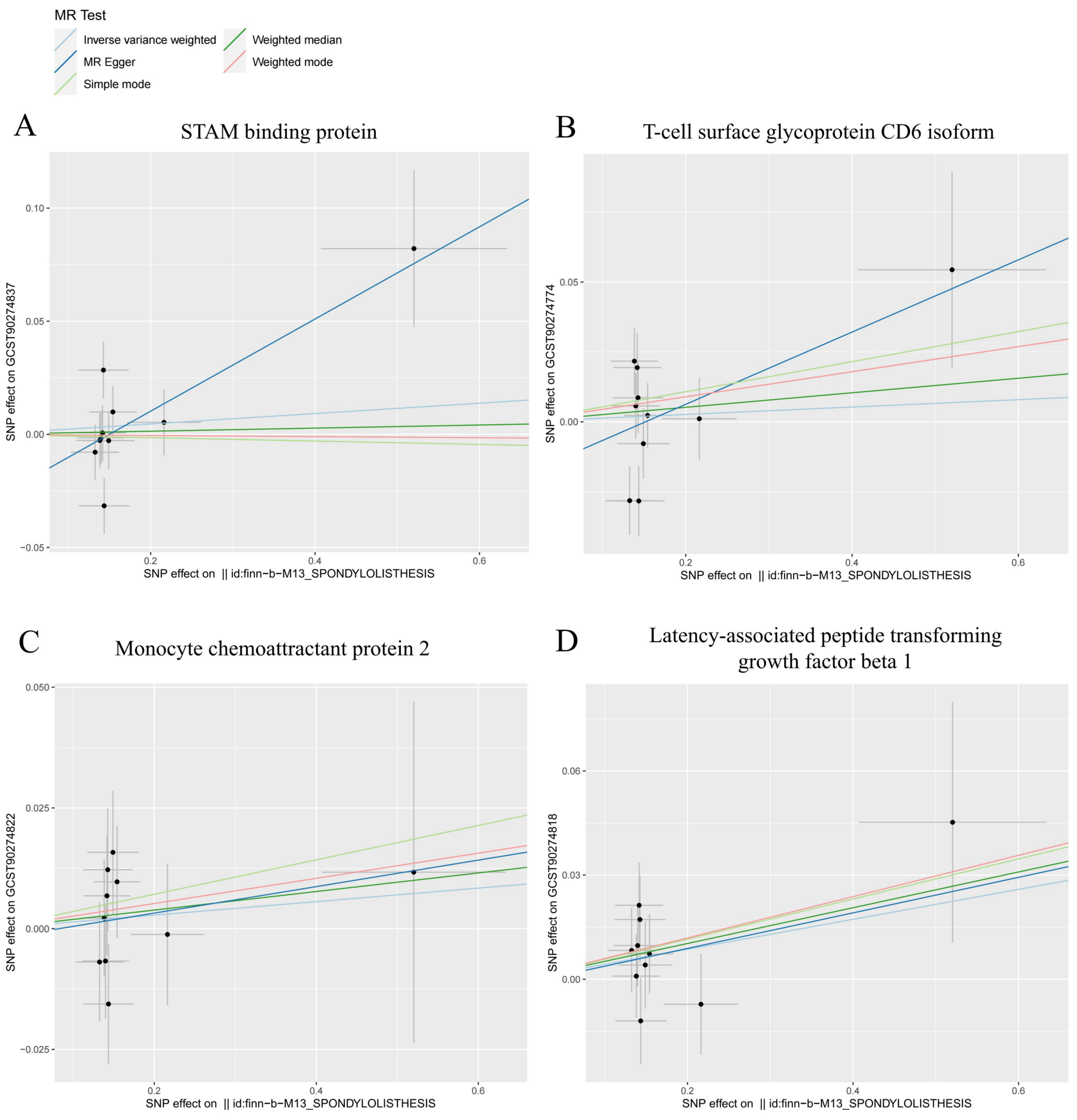

Figure S21. Scatter plots of causal associations between exposure (spondylolisthesis/spondylolysis) and outcomes (CIPs). (A) Scatter plot between spondylolisthesis/spondylolysis and STAM binding protein; (B) Scatter plot between spondylolisthesis/spondylolysis and T-cell surface glycoprotein CD6 isoform; (C) Scatter plot between spondylolisthesis/spondylolysis and monocyte chemoattractant protein 2; (D) Scatter plot between spondylolisthesis/spondylolysis and latency-associated peptide transforming growth factor beta 1.

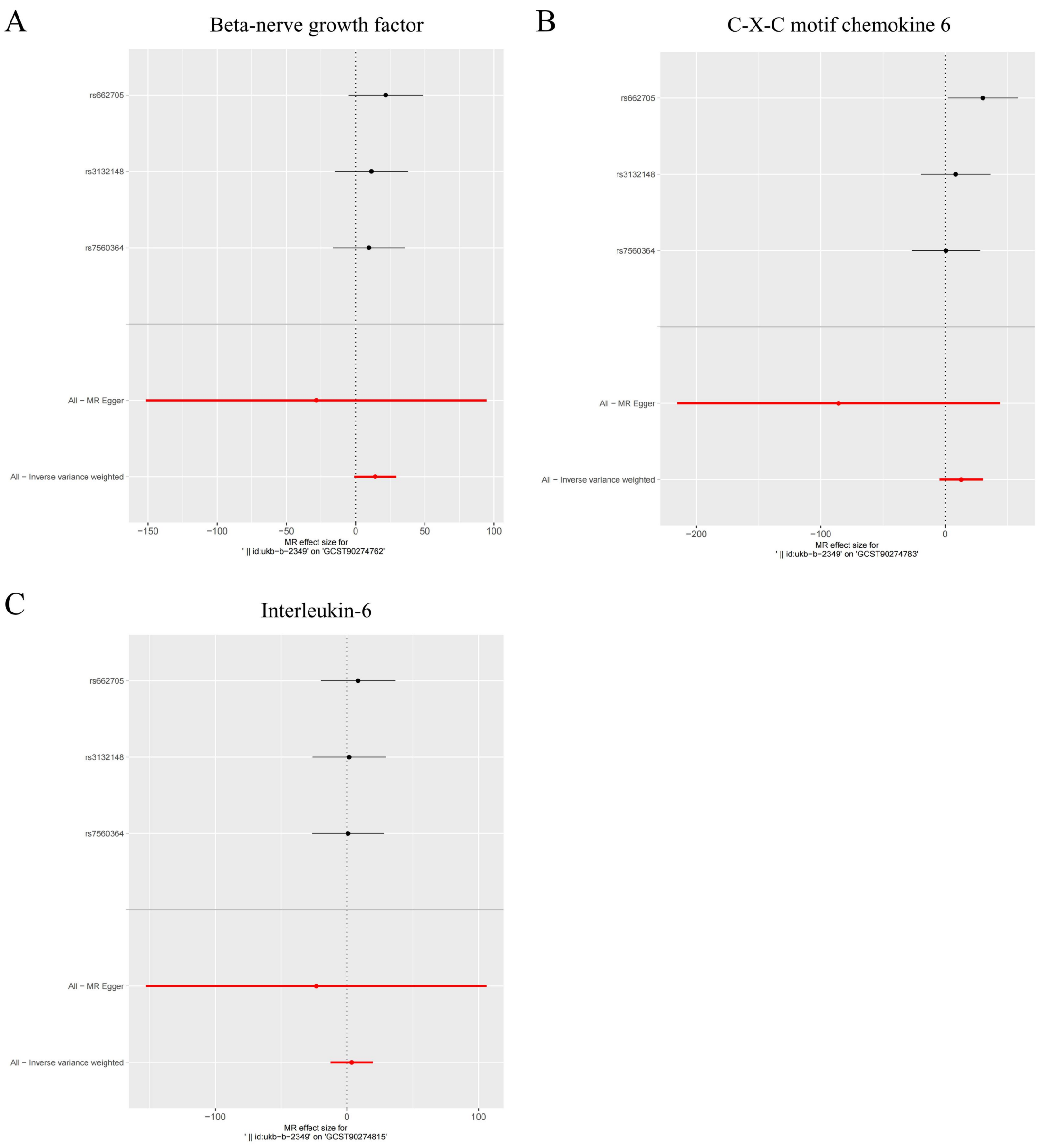

Figure S22. Forest plots of causal associations between exposure (CS) and outcomes (CIPs). (A) Forest plot between CS and beta-nerve growth factor; (B) Forest plot between CS and C-X-C motif chemokine 6; (C) Forest plot between CS and interleukin-6.

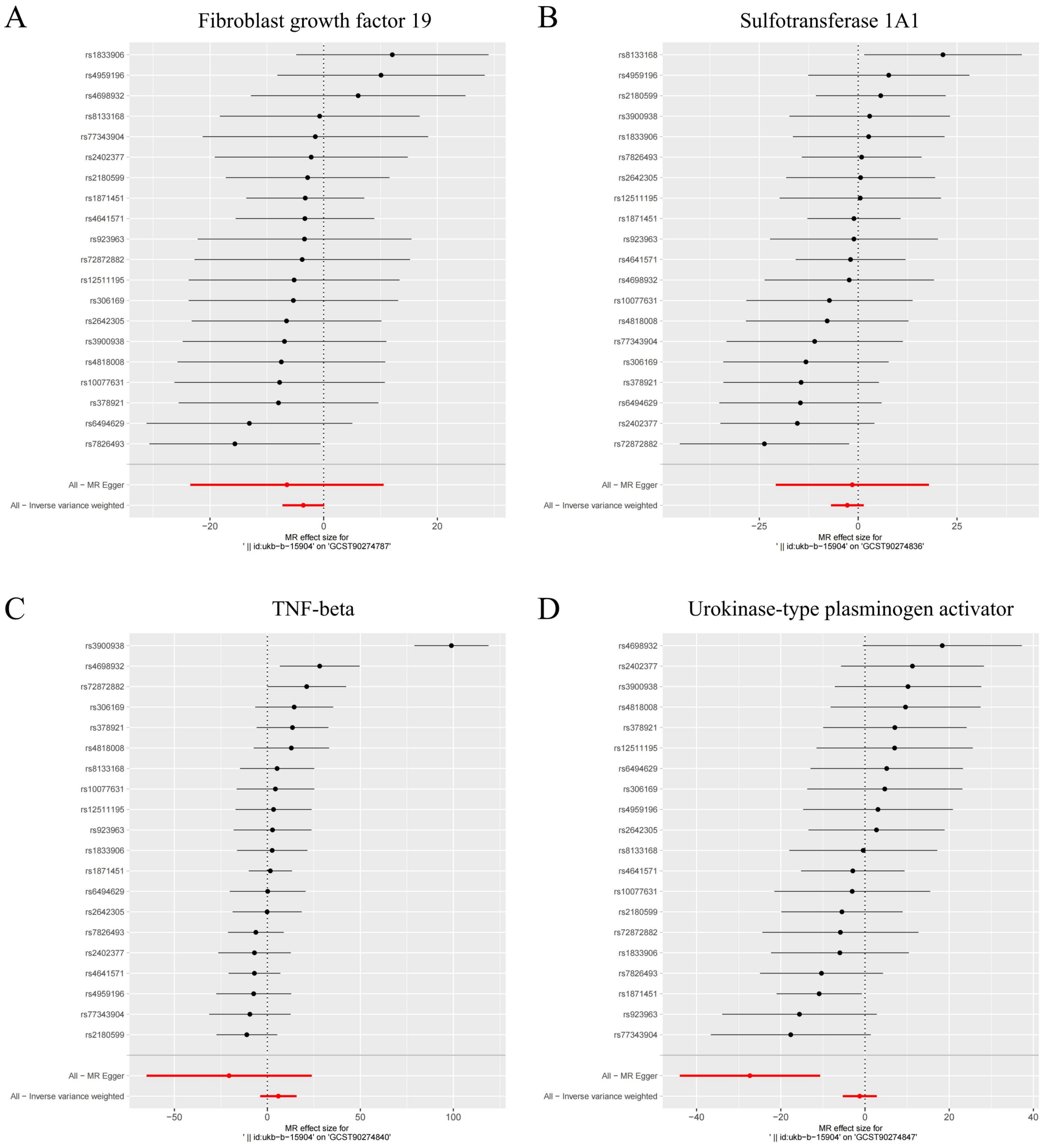

Figure S23. Forest plots of causal associations between exposure (PD/SD) and outcomes (CIPs). (A) Forest plot between PD/SD and fibroblast growth factor 19; (B) Forest plot between PD/SD and sulfotransferase 1A1; (C) Forest plot between PD/SD and TNF-beta; (D) Forest plot between PD/SD and urokinase-type plasminogen activator.

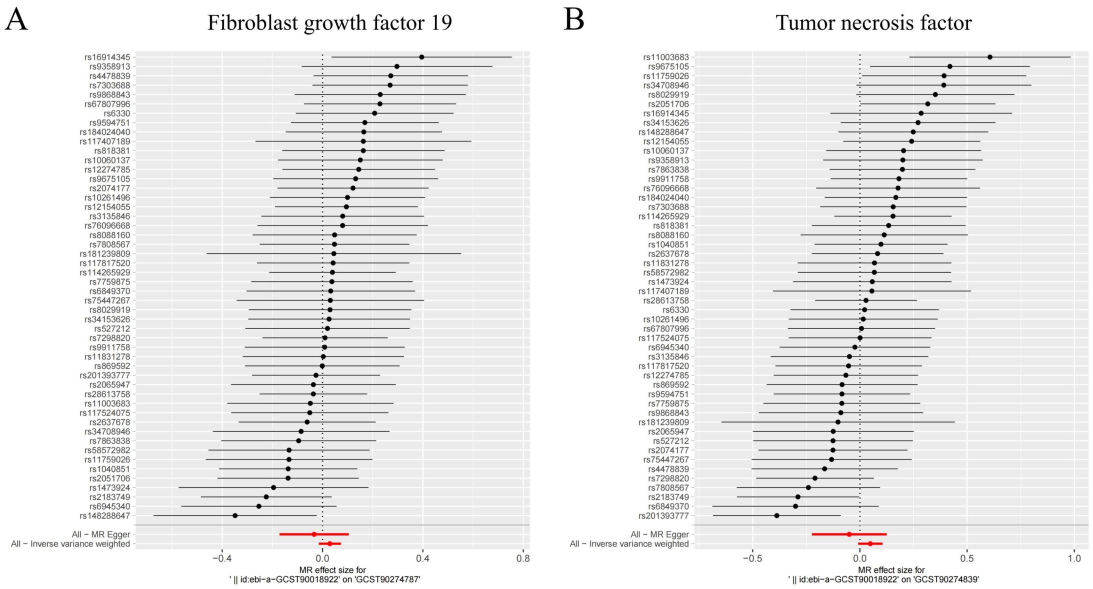

Figure S24. Forest plots of causal associations between exposure (SCS) and outcomes (CIPs). (A) Forest plot between SCS and fibroblast growth factor 19; (B) Forest plot between SCS and tumor necrosis factor.

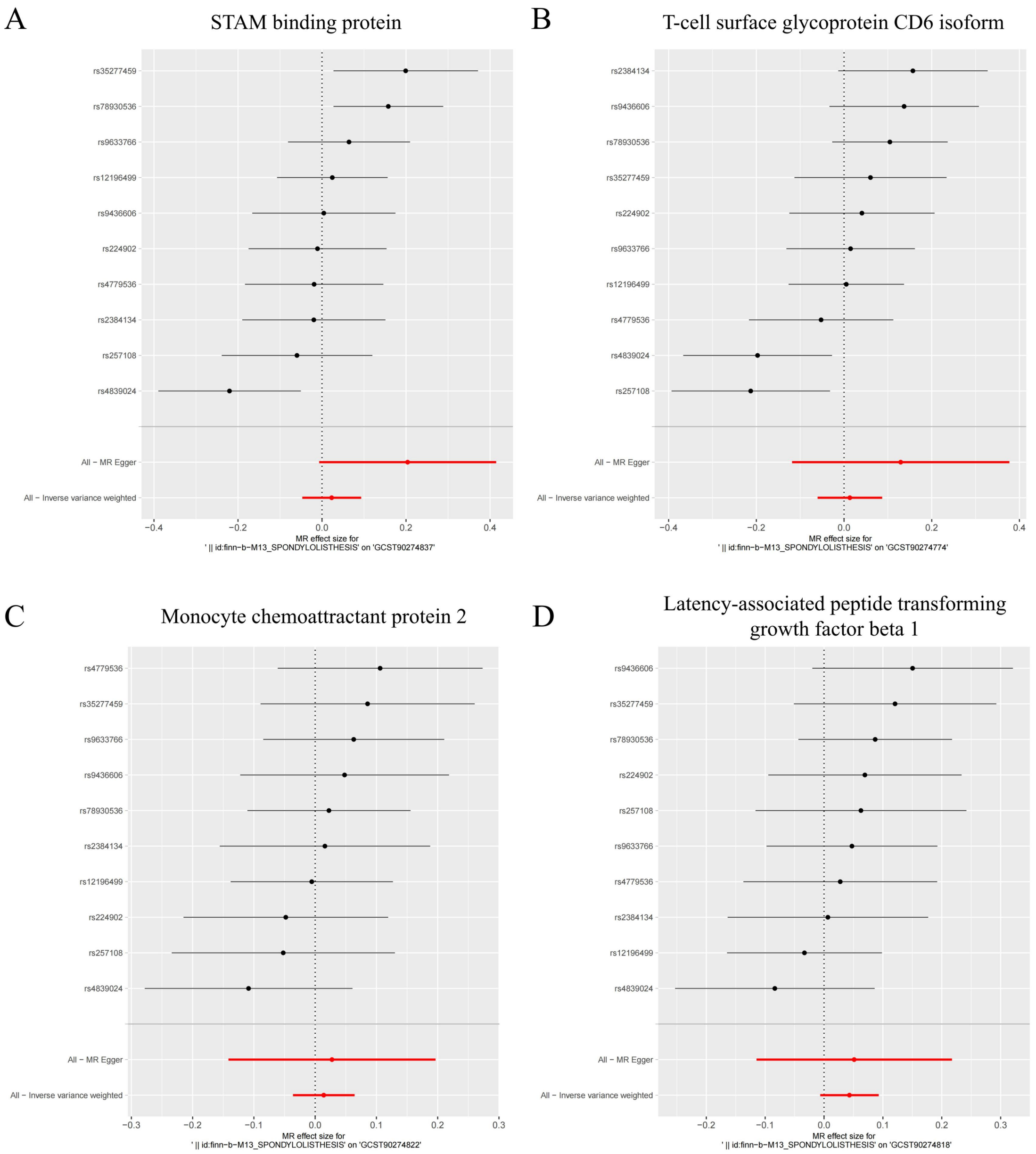

Figure S25. Forest plots of causal associations between exposure (spondylolisthesis/spondylolysis) and outcomes (CIPs). (A) Forest plot between spondylolisthesis/spondylolysis and STAM binding protein; (B) Forest plot between spondylolisthesis/spondylolysis and T-cell surface glycoprotein CD6 isoform; (C) Forest plot between spondylolisthesis/spondylolysis and monocyte chemoattractant protein 2; (D) Forest plot between spondylolisthesis/spondylolysis and latency-associated peptide transforming growth factor beta 1.

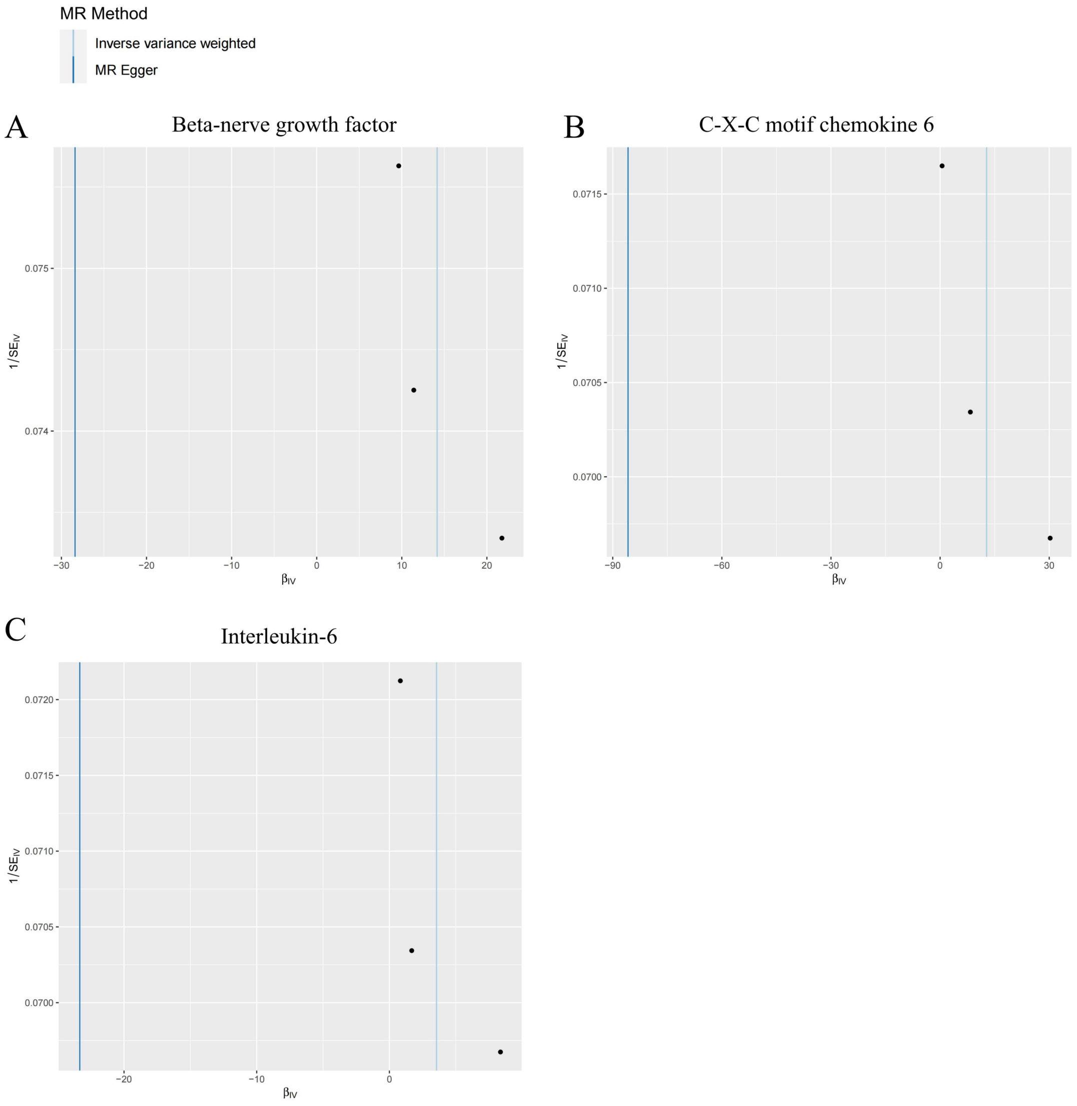

Figure S26. Funnel plots of causal associations between exposure (CS) and outcomes (CIPs). (A) Funnel plot between CS and beta-nerve growth factor; (B) Funnel plot between CS and C-X-C motif chemokine 6; (C) Funnel plot between CS and interleukin-6.

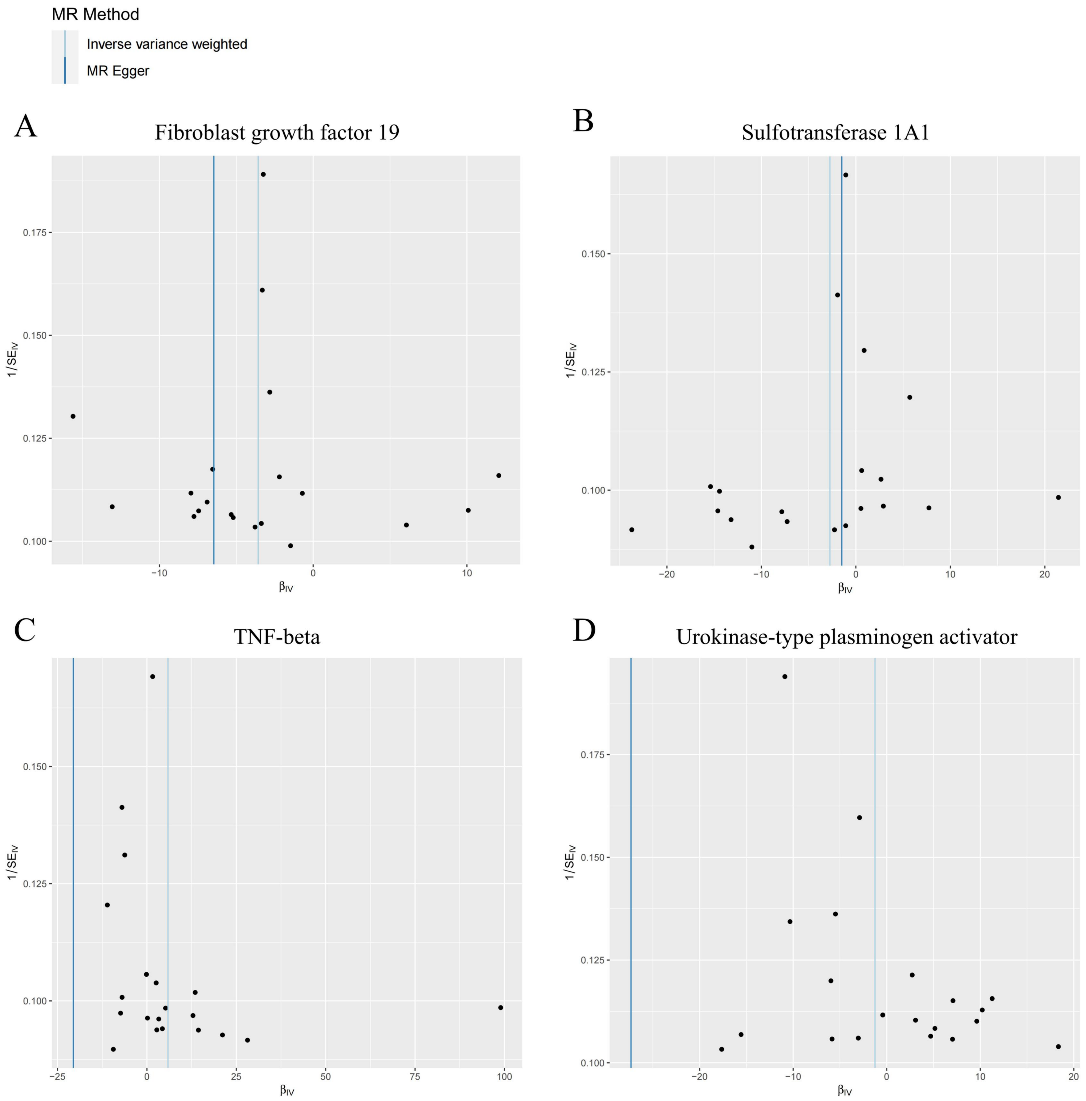

Figure S27. Funnel plots of causal associations between exposure (PD/SD) and outcomes (CIPs). (A) Funnel plot between PD/SD and fibroblast growth factor 19; (B) Funnel plot between PD/SD and sulfotransferase 1A1; (C) Funnel plot between PD/SD and TNF-beta; (D) Funnel plot between PD/SD and urokinase-type plasminogen activator.

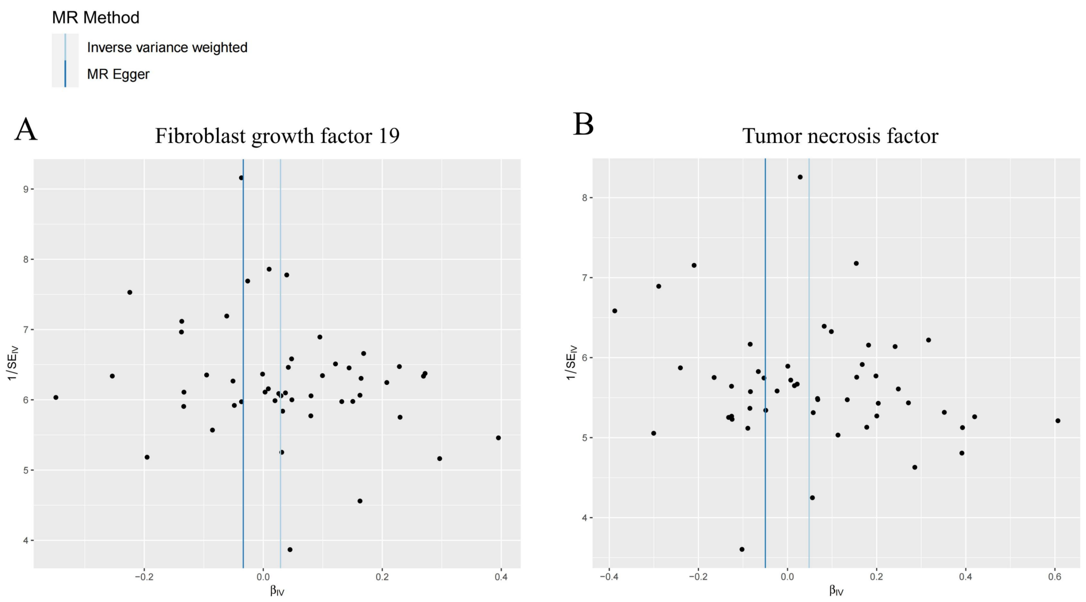

Figure S28. Funnel plots of causal associations between exposure (SCS) and outcomes (CIPs). (A) Funnel plot between SCS and fibroblast growth factor 19; (B) Funnel plot between SCS and tumor necrosis factor.

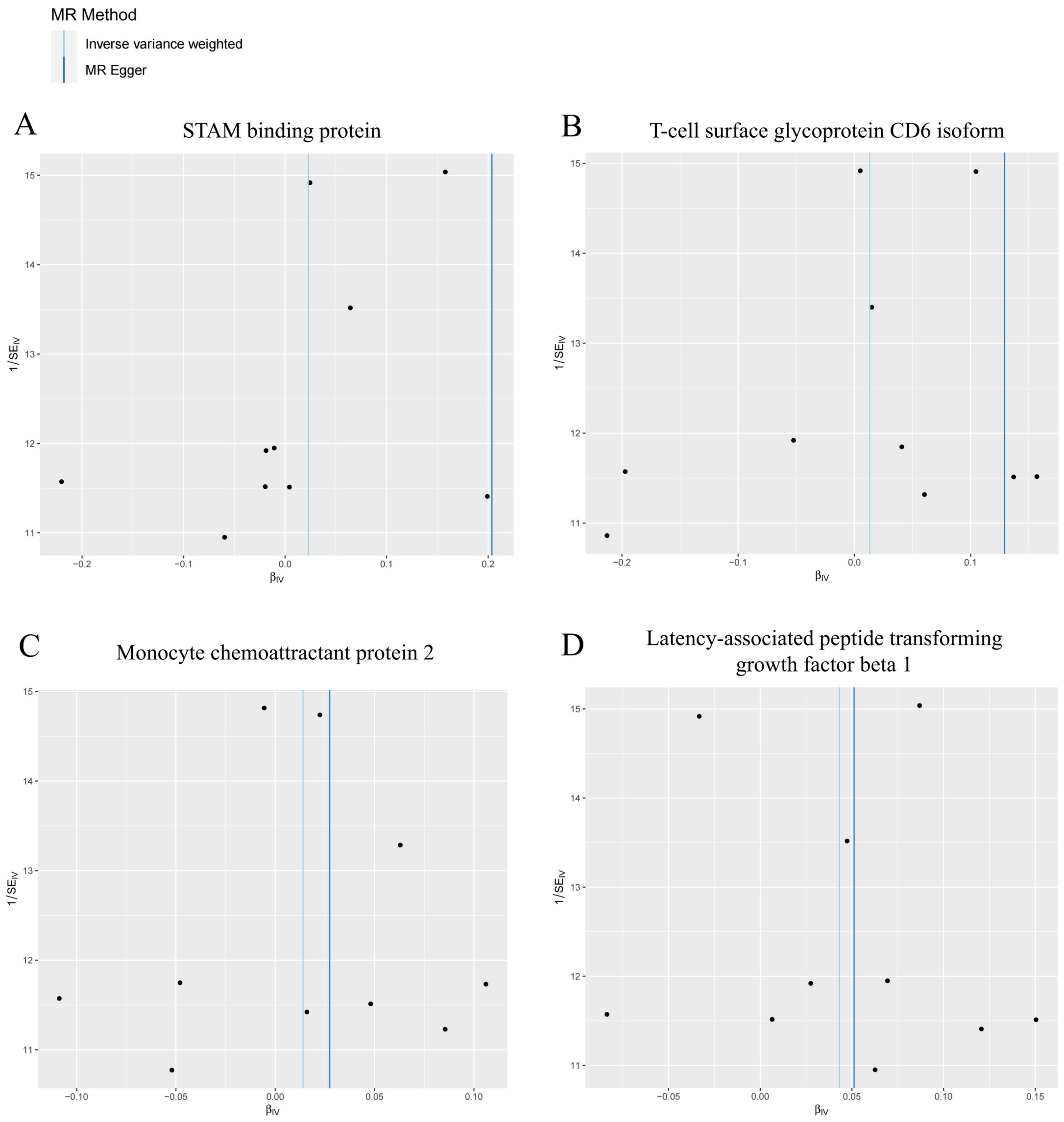

Figure S29. Funnel plots of causal associations between exposure (spondylolisthesis/spondylolysis) and outcomes (CIPs). (A) Funnel plot between spondylolisthesis/spondylolysis and STAM binding protein; (B) Funnel plot between spondylolisthesis/spondylolysis and T-cell surface glycoprotein CD6 isoform; (C) Funnel plot between spondylolisthesis/spondylolysis and monocyte chemoattractant protein 2; (D) Funnel plot between spondylolisthesis/spondylolysis and latency-associated peptide transforming growth factor beta 1.

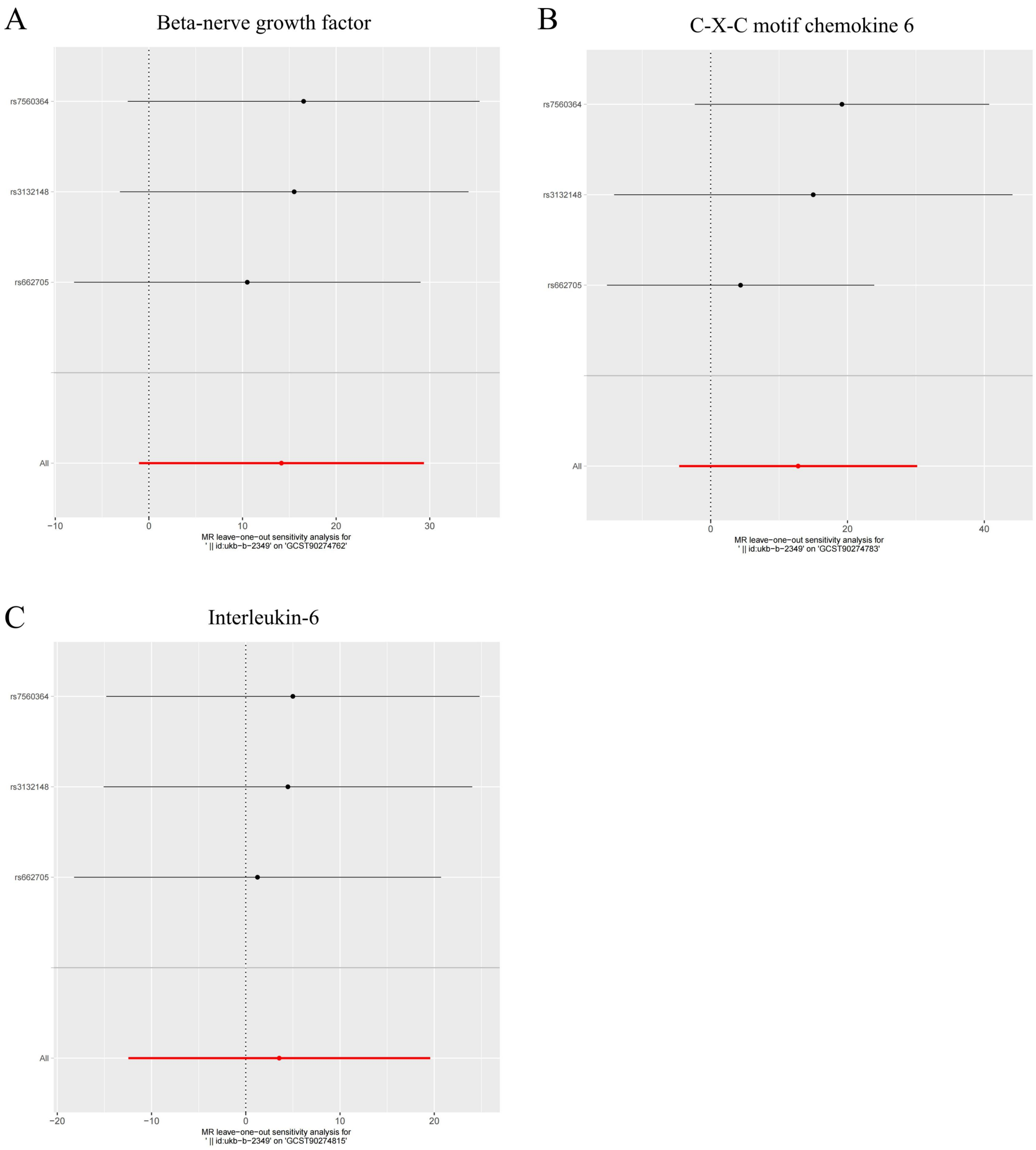

Figure S30. Leave-on-out plots of causal associations between exposure (CS) and outcomes (CIPs). (A) Leave-on-out plot between CS and beta-nerve growth factor; (B) Leave-on-out plot between CS and C-X-C motif chemokine 6; (C) Leave-on-out plot between CS and interleukin-6.

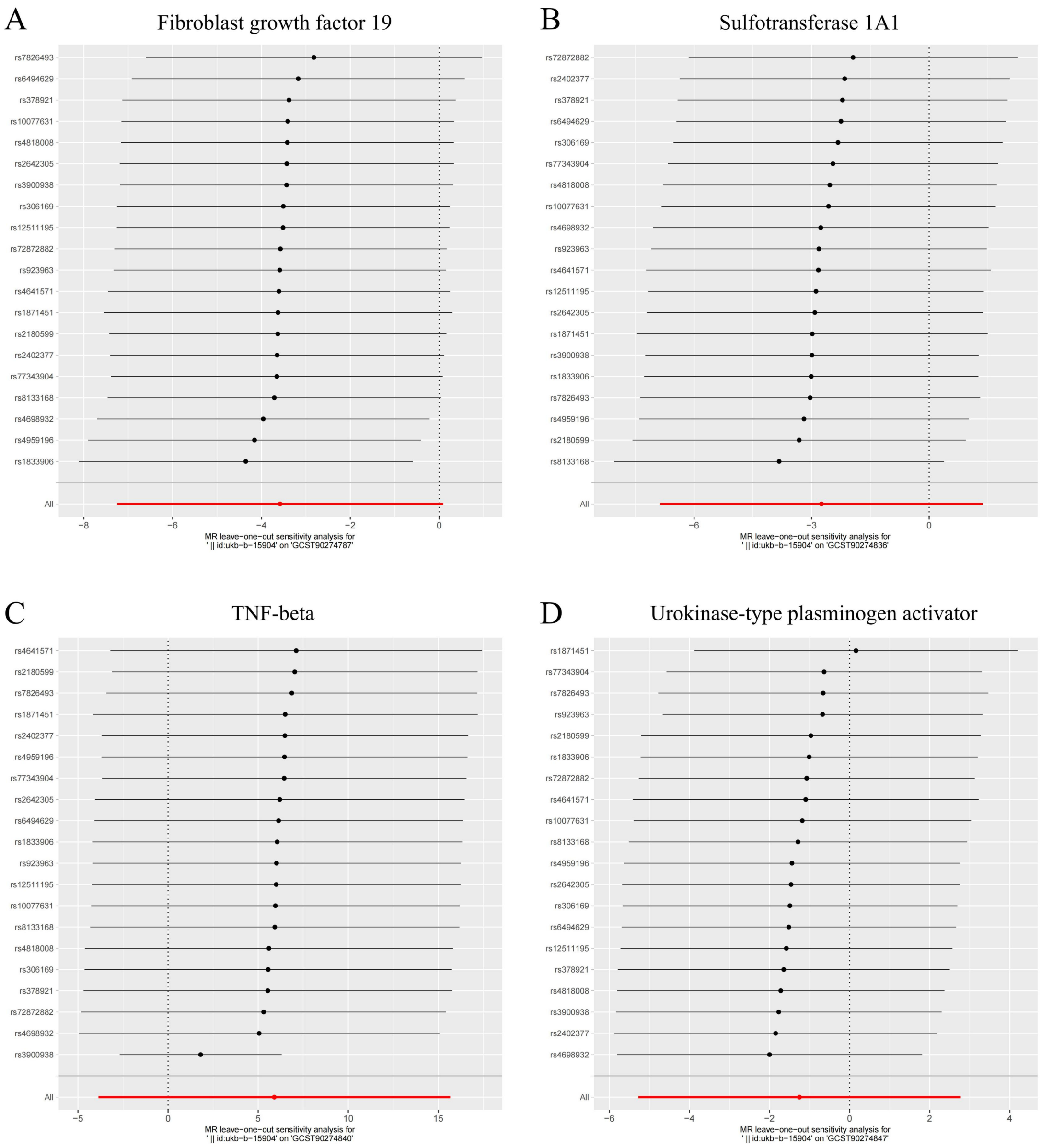

Figure S31. Leave-on-out plots of causal associations between exposure (PD/SD) and outcomes (CIPs). (A) Leave-on-out plot between PD/SD and fibroblast growth factor 19; (B) Leave-on-out plot between PD/SD and sulfotransferase 1A1; (C) Leave-on-out plot between PD/SD and TNF-beta; (D) Leave-on-out plot between PD/SD and urokinase-type plasminogen activator.

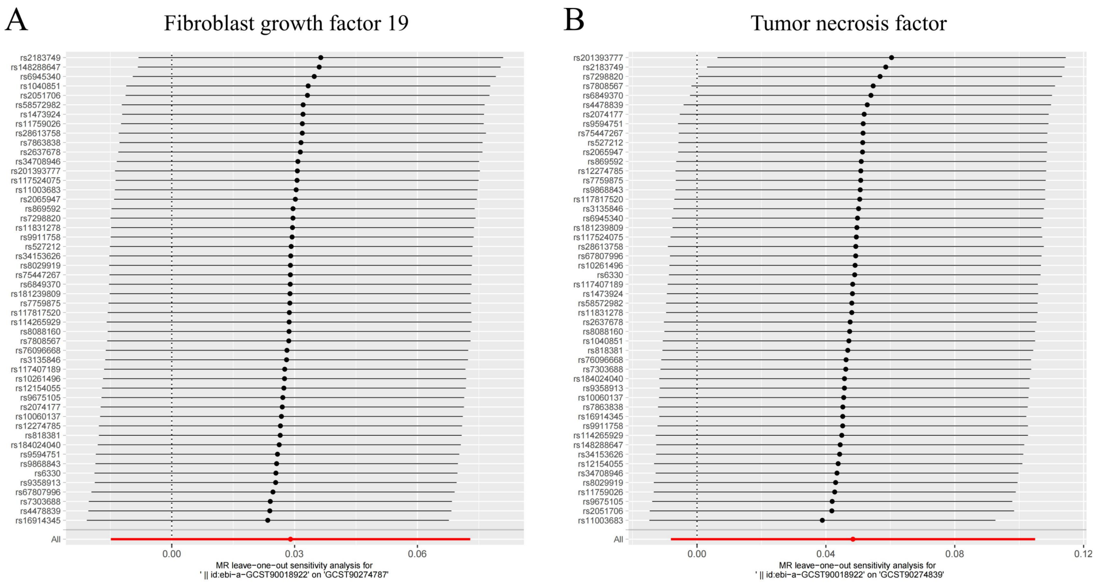

Figure S32. Leave-on-out plots of causal associations between exposure (SCS) and outcomes (CIPs). (A) Leave-on-out plot between SCS and fibroblast growth factor 19; (B) Leave-on-out plot between SCS and tumor necrosis factor.

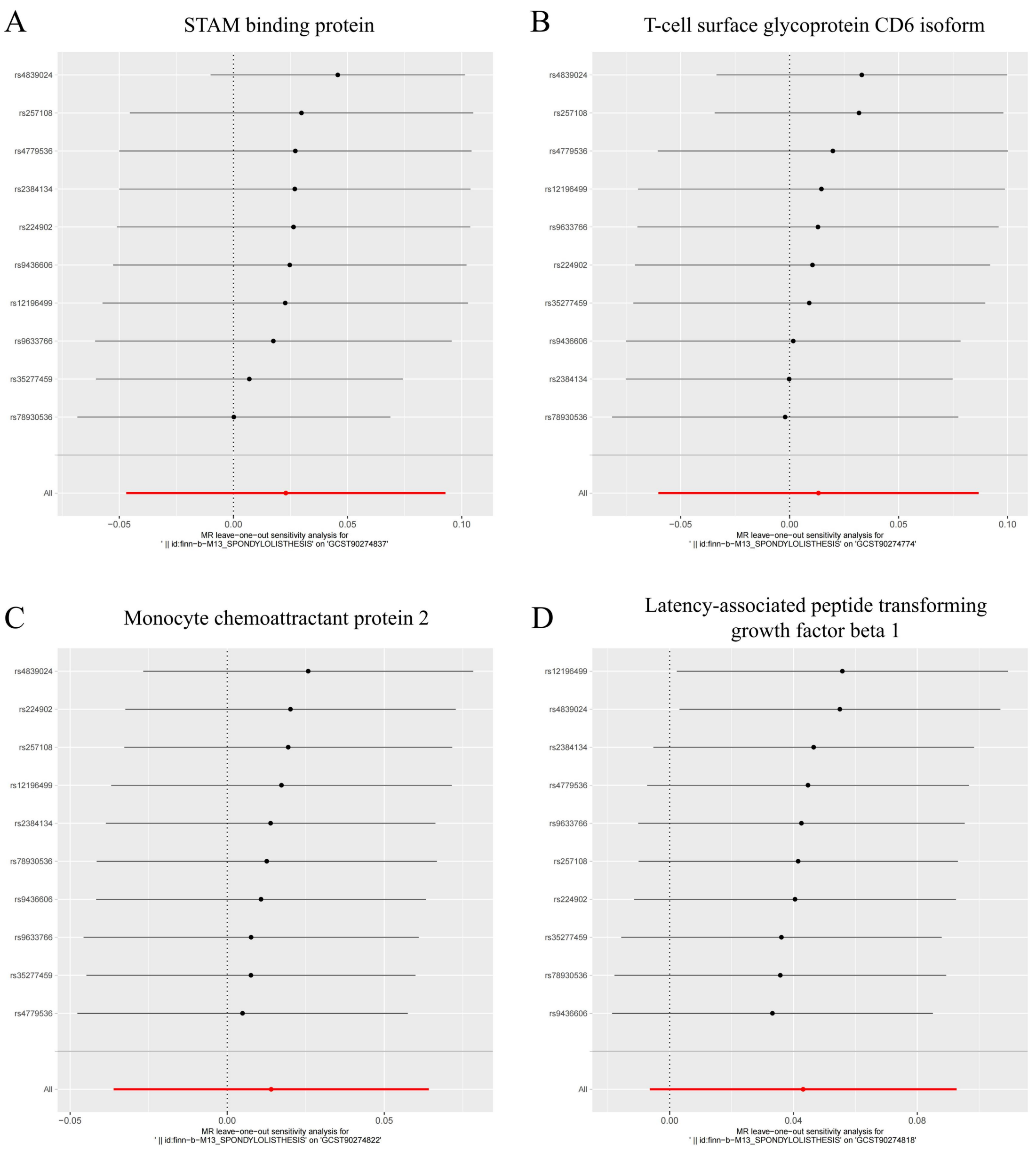

Figure S33. Leave-on-out plots of causal associations between exposure (spondylolisthesis/spondylolysis) and outcomes (CIPs). (A) Leave-on-out plot between spondylolisthesis/spondylolysis and STAM binding protein; (B) Leave-on-out plot between spondylolisthesis/spondylolysis and T-cell surface glycoprotein CD6 isoform; (C) Leave-on-out plot between spondylolisthesis/spondylolysis and monocyte chemoattractant protein 2; (D) Leave-on-out plot between spondylolisthesis/spondylolysis and latency-associated peptide transforming growth factor beta 1.
